# Structural Studies of Piperine Inclusion Complexes in Native and Derivative β-Cyclodextrins

**DOI:** 10.3390/biom12121762

**Published:** 2022-11-26

**Authors:** Elias Christoforides, Athena Andreou, Andreas Papaioannou, Kostas Bethanis

**Affiliations:** 1 Physics Laboratory, Department of Biotechnology, Agricultural University of Athens, 75 Iera Odos, 11855 Athens, Greece; 2 Department of Biomedical Sciences, University of West Attica, Campus 1, Ag. Spyridonos 28, 12243 Athens, Greece; 3 Genetics Laboratory, Department of Biotechnology, Agricultural University of Athens, 75 Iera Odos, 11855 Athens, Greece

**Keywords:** piperine, *β*-cyclodextrin, methylated *β*-cyclodextrin, 2-hydroxypropyl-*β*-cyclodextrin, inclusion complex, molecular dynamics, phase solubility, X-ray crystallography

## Abstract

Piperine (PN), the primary pungent alkaloid in black pepper shows several biological activities such as antioxidant, antimicrobial and anti-cancerogenic effects. Similar to other alkaloids, PN is characterized by poor water solubility. One way to improve its solubility and thus its biological activities is by forming inclusion complexes with suitable cyclodextrins. In this work PN inclusion complexes in native *β-*cyclodextrin (*β-*CD), its methylated (randomly methylated (RM-*β-*CD), heptakis-(2,6-di-*O*-methyl)-*β*-CD (DM-*β-*CD) and heptakis-(2,3,6-tri-*O*-methyl)-*β*-CD (TM-*β-*CD)) and 2-hydroxypropylated (HP-*β-*CD) derivatives are investigated using physicochemical methods, such as phase solubility study and X-ray crystallography complemented by theoretical (molecular dynamics simulations) studies. The determination of the crystal structure of the PN inclusion complexes in *β-*CD, DM-*β-*CD and TM-*β-*CD, reveals the formation of 1:2 guest:host inclusion complexes in the crystalline state. The guest PN molecule threads the hydrophobic cavities of the hosts which are arranged as couples in a tail-to-tail mode in the case of PN/*β-*CD and in a head-to-tail mode in the cases of PN/DM-*β-*CD and PN/TM-*β-*CD. MD studies based on the crystallographically determined structures and docked models show the stability of the examined complexes in an aqueous environment whereas the binding affinity of PN for the host molecules is calculated by the MM/GBSA method. Finally, phase-solubility studies of PN with *β-*CD, RM-*β-*CD and HP-*β-*CD are presented, indicating a B_s_-type for the PN/*β-*CD complex and an A_L_-type for the PN/RM-*β-*CD and PN/HP-*β-*CD complexes with 1:1 guest:host stoichiometry.

## 1. Introduction

**Figure 1:**
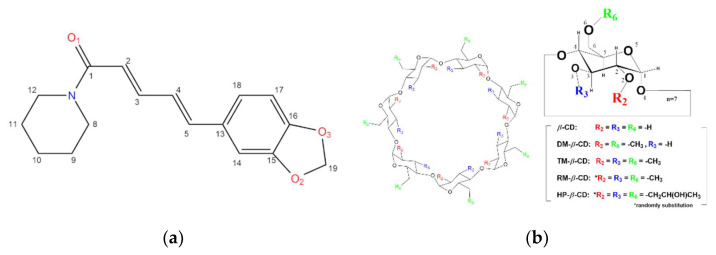
Schematic representation of the chemical structure of (**a**) Piperine (PN); (**b**) *β*-cyclodextrin (*β-*CD), heptakis(2,6-di-*O*-methyl)-*β*-cyclodextrin (DM-*β-*CD), heptakis(2,3,6-tri-*O*-methyl)-*β*-cyclodextrin (TM-*β-*CD), randomly-methylated-*β*-cyclodextrin (RM-*β-*CD) and 2-hydroxypropyl-*β*-cyclodextrin (HP-*β-*CD).

Black pepper (*Piper nigrum*) is one of the most important spices worldwide due to its many biological properties such as antioxidant, antimicrobial, anti-inflammatory, and anticancer activities [1]. Its characteristic pungent taste and flavor is attributed to the presence of the alkaloid piperine (1-(5-[1,3-benzodioxol-5-yl]-1-oxo-2,4-pentadienyl) piperidine), for short PN, Figure 1a). Although piperine is consumed as a dietary spice and has been considered as functional food [2], it has also many pharmaceutical benefits. More specifically, piperine is known as a phytochemical and antimicrobial [3] or antifungal agent acting as an inhibitor for certain enzymes [4], but it also affects the activity of catalase and glutathione peroxidase enzymes [5] preventing in this way the outbreak of negative biological procedures and diseases like Parkinson and Alzheimer [6,7]. However, its ability to reduce the risk of developing certain cancers [8] and to exhibit anticarcinogenic effects [9] have recently drawn great attention. Piperine can reverse multidrug resistance (MDR) in cancer cells and acts as a bioavailability enhancer for many chemotherapeutic agents [10], as many studies indicate that piperine shows synergistic effects when taken in combination with various classes of drugs [11].

However, its potential application in functional foods and pharmaceutical formulations is incommoded as piperine is practically insoluble in water, slightly soluble in other permissible pure solvents [12] and unstable in ultraviolet light [13]. An already tested approach, in order to improve the physicochemical properties of piperine (i.e., increase its water solubility and protect it from degradation) and thus effectively enhance its bioavailability and activity, is by the formation of piperine inclusion complexes in suitable cyclodextrins (CDs) [14,15]. CDs are amphiphilic cyclic oligosaccharides consisting of at least six D-(+) glucopyranose units attached by α-(1, 4) glycosidic bonds. Their distinctive round conformation of a truncated cone facilitates the encapsulation of certain guest molecules inside their interior cavity. Beta-cyclodextrin (*β*-CD), which is comprised of seven glucose units is the most common representative in food and pharmaceutical industries due to its suitable cavity size for the accommodation of several food ingredients or drugs, and its low cost. The hydroxyl groups of its macrocycle rims participate in several intra- and intermolecular (host-host and host-guest) hydrogen bonds. Methylation of these hydroxyls in both rims results in its methylated derivatives that are characterized by significantly higher water solubility and flexibility compared to the parental *β*-CDs. (Heptakis(2,6-di-O-methyl)-*β*-cyclodextrin (DM-*β*-CD), heptakis(2,3,6-tri-O-methyl)-*β*-cyclodextrin (TM-*β-*CD) and randomly methylated *β*-cyclodextrin (RM-*β-*CD) (Figure 1b). Another *β*-CD derivative, obtained by substituting the aforementioned hydroxyls with 2-hydroxypropyl groups, is the 2-hydroxypropyl-*β*-Cyclodextrin (HP-*β*-CD), which is also well known for its pharmaceutical applications (Figure 1b). CDs have a distinct role in the pharmaceutical industry, either by their direct use as drugs [16] or as drug carriers participating in nanoparticle (NPs) formulations [17].

The inclusion of piperine in native *β*-CD has been studied by various spectroscopic (phase solubility studies, Job’s Plot, FT-IR, near-infrared spectroscopy (NIR), Raman, ^1^H-NMR, UV-visible absorption and fluorescence intensity study, powder X-ray diffraction (PXRD)) and calorimetric (differential scanning calorimetry (DSC) and thermogravimetry (TG)) methods as well as by scanning electron microscopy (SEM) [18,19,20,21,22,23]. The above methods indicated the formation of PN/*β*-CD complexes of either 1:1 or 1:2 guest:host stoichiometry, according to the interactions found between the aromatic ring of PN and *β*-CD, with complexation efficiency varying from 7 to 70% for various stoichiometric ratios. Moreover, the dissolution rate behavior of the complex was examined via dissolution testing assays. Although in the above-mentioned works, the inclusion complex has been also studied in the solid state, no crystal structure of piperine complexes, either in native *β*-CD or in *β*-CD derivatives has been presented so far. In this work, the inclusion compounds of piperine in *β*-CD, DM-*β*-CD and TM-*β-*CD are investigated by single crystal X-ray diffraction (SC-XRD), revealing the stoichiometry, interactions and geometrical details of the complex in the crystalline state. Based on the crystallographically determined coordinates, molecular dynamics (MD) studies have also been performed in order to monitor the dynamic behavior and the stability of the complexes in aqueous environments and in the absence of crystal contacts. Finally, phase solubility studies in aqueous solution were carried out for PN/*β*-CD, PN/RM-*β-*CD and PN/HP-*β-*CD inclusion complexes, in order to examine the solubility profile and estimate the apparent stability constant (K_1:1_) and the compexation efficiency (CE) for these complexes. In the case of PN/HP-*β-*CD, where no crystal structure is available, the 1:1 guest:host stoichiometry, indicated by its solubility profile, was used for the preparation of the docked model which was further examined by MDs.

The complementary structural analysis by experimental (X-ray crystallography) and theoretical (MD) studies presented in this work, sheds light on the structure-stability relationship of the examined “spicy” cyclodextrin inclusion complexes. Our understanding of the structural aspects of these complexes may be useful in the engineering of modified guest-host preparations with optimized pharmacological properties and shape future piperine applications [24].

## 2. Materials and Methods

### 2.1. Materials

Piperine (97% pure) as a light yellow powder was purchased from Merck KGaA (Damstadt, Germany), while *β*-CD, DM-*β*-CD, TM-*β*-CD, RM-*β*-CD (degree of substitution (DS) ~12) and HP-*β*-CD (DS~4.5) of pharmaceutical grade quality as white powders were from Cyclolab Ldt. (Budapest, Hungary). Double distilled water was utilized for the preparation of all the examined solutions.

### 2.2. Phase Solubility Study

The UV–visible (UV–Vis) spectrophotometer BK-S380 (BioBase Group, Jinan, Shandong, China) was utilized for all spectroscopic analyses concerning the solubility of pure PN and the phase solubility studies. The calibration curve was obtained at the visible absorption maximum of the PN (345 nm) as follows: Five standards of PN solution in a 1:1 methanol: water ratio corresponding to concentrations of 0.00125, 0.0025, 0.005, 0.01 and 0.02 mM were prepared and measured at 345 nm. The calibration curve was depicted by plotting the absorbance against the above PN concentrations and by applying the linear regression analysis according to a previously described procedure [25]. Subsequently, phase solubility studies were carried out according to the method reported by Higuchi and Connors [26]. More explicitly, an excess amount of PN (50 mg) was added to 10 mL of deionized water containing various concentrations between 1 to 15 mM for *β*-CD and 1 to 60 mM for both RM-*β*-CD and HP-*β*–CD. The solutions were further mixed using an orbital shaker (PHOENIX Instrument Laboratory Shaker RS-OS 5, Berlin, Germany) at 25 °C for 48 h to ensure equilibrium, and then the solutions were passed through a 0.45-μm filter to remove the undissolved solids. The filtered solutions were appropriately diluted with a 1:1 (*v*/*v*) methanol: water solution and measured at 345 nm.

### 2.3. Single-Crystal Preparation 

In the crystal formation process of native CD supramolecular complexes, the slow cooling method, where the temperature of an aqueous saturated mixture solution of CD and the guest molecule is gradually decreased from 343 K to ambient temperature, was followed. More specifically, 20 mg of *β*-CD (0.0175 mmoles) were weighted into vials and 2 mL of distilled water was added. An equimolar quantity (5.0 mg, 0.0175 mmoles) of PN was added and the mixture was stirred for about four hours at 343 K until it was limpid. Clear light colourless prismatic-like crystals suitable for X-ray data measurements were obtained over a seven-day period. 

On the other hand, the slow evaporation method, which is more suitable for crystallizing inclusion complexes of methylated CDs, was used in the cases of DM-*β-*CD and TM-*β-*CD inclusion complexes. Briefly, a suitable amount of PN was added to aqueous solutions of DM-*β-*CD or TM-*β-*CD (0.004 M) at 1:1 host: guest mole ratios. The two mixtures were stirred for 1 h at room temperature and subsequently maintained at 321 K for a period of one week. Clear light colourless rod-like and prism-like crystals, suitable for X-ray data collection, were obtained in the case of PN/DM-*β-*CD and PN/TM-*β-*CD, respectively.

### 2.4. X-ray Diffraction Experiments 

Data collection was performed using CuK*a* radiation (λ = 1.54178 Å) in a Bruker D8-VENTURE diffractometer equipped with the CMOS-based detector PHOTON 100. The tested specimens were harvested from the mother liquor, cryo-protected by rapid immersion in paraffin oil and flash frozen using a continuous nitrogen-flow cooling device (Oxford Cryosystems Ltd., Long Handorough, UK) at 100 or 120 K.

The data were processed with the Bruker SAINT Software package [27] using a narrow-frame algorithm and were corrected for absorption effects using the multi-scan method (SADABS) [28].

In the case of the PN/*β-*CD complex, a total of 2494 frames were collected in a total exposure time of 15.1 h. The crystal was twinned. Based on a determined triclinic unit cell, two main domains were detected related by a rotation angle of 179.91°. The integration of the images using both domains and the subsequent scaling of the data by using the TWINABS program [29] yielded 16,593 and 17,055 total reflections with I/σ(I) of 33.0 and 33.2 for domain 1 and 2, respectively, whereas 17,875 reflections were attributed as composites (overlapped reflections from domain 1 and 2). The twin fraction was close to 0.5. The resolution of the measured data was up to 0.84 Å. However, due to the low completeness of the measured data set (94.4%), a lower resolution limit of 0.86 Å with a completeness of 98%, was used for the refinement of the crystal structure. The final cell constants and refinement details are listed in Table 1.

The X-ray diffraction from a crystal of PN/DM-*β-*CD complex, resulted in a total of 2860 frames which were collected in 21 h. A monoclinic unit cell of *P*2_1_ space group symmetry was defined and the integration procedure yielded a total of 59,020 reflections to a maximum θ angle of 63.89° (0.86 Å resolution), of which 12,372 were independent (average redundancy 4.770, completeness 99.1%, R_int_ = 7.19%, R_sig_ = 5.26%) and 10,678 (86.31%) were greater than 2σ(F^2^). The final cell constants, quoted in Table 1, are based upon the refinement of the XYZ-centroids of 9685 reflections above 20 σ(I) with 7.934° < 2θ < 128.5°.

Finally, in the case of the crystal of PN/TM-*β-*CD complex, a total of 1706 frames were collected in 14 h. The unit cell was also a monoclinic *P*2_1_ and the integration of the collected data yielded a total of 73,723 reflections to a maximum θ angle of 50.66° (1.00 Å resolution). The final cell constants, based upon the refinement of the XYZ-centroids of 9801 reflections above 20 σ(I) with 6.544° < 2θ < 100.4°, are also quoted in Table 1 along with other refinement information. 

The structure solution of these CD complexes was based on the Patterson-seeded dual-space recycling utility of the SHELXD program [30]. The structures were refined by full-matrix least squares against *F*^2^ by using SHELXL-2014/7 [31] in the SHELXLE GUI [32]. Due to the limited resolution, structural complexity and disorder of the final models, soft restraints on bond lengths and angles, generated from the PRODRG2 webserver [33], were applied on the host and guest molecules of the inclusion complexes. Anisotropic displacement parameters were refined using soft restraints (SIMU) [34] implemented in the *SHELXL* program where necessary.

All hydrogen atoms were placed in geometric positions and treated as riding on their parent atoms with *d*_C–H_ = 0.95–1.00 Å (depending on the hybridization of carbon atom) and *d*_O–H_ = 0.84 Å. *U*iso(H) values were assigned in the range 1.2–1.5 times *U*eq of the parent atom. Hydrogen atoms of water molecules were not included in any of the final structural models. In an effort to maintain a relatively high (>6.0) data/parameters ratio, anisotropic thermal parameters were imposed to selected, non-H atoms of the host molecules. Extinction corrections were applied to the PN/DM-*β-*CD case, while 8, 33 and 12 reflections that exhibited poor agreement to the refined models were omitted in the PN/*β-*CD, PN/DM-*β-*CD and PN/DM-*β-*CD cases, respectively.

The programs Mercury [35], Pymol [36] and Olex2 [37] were used to explore the crystal packing, analyze the structure geometry and visualize the asymmetric unit, the intermolecular interactions and the crystal packing of the complexes. Crystallographic data are given in Table 1. Crystallographic information files with embedded structure factors have been checked and validated for the consistency and integrity of crystal structure determinations according to IUCr standards and have been deposited in the Cambridge Structural Database (CSD) under the deposition numbers CCDC: 2063126, 2022888 and 2063127.

### 2.5. Computational Methods

Five separate MD simulations involving piperine inclusion complexes with the native and three modified *β*-CDs, illustrating different guest:host ratios and different inclusion modes were carried out in an effort to monitor their time-resolved motions in aqueous media. For the PN/*β*-CD complex, the crystallographically determined atomic coordinates of four adjacent *β-*CD molecules (two of them forming a dimer) and two encapsulated guests (PN1 and PN2) inside their cavity comprise the staring model of a 2:4 guest:host stoichiometry. For the inclusion complexes of piperine in methylated CDs, PN/DM-*β*-CD and PN/TM-*β*-CD, the initial models were also based on the crystallographically determined atomic coordinates (both with a 1:2 guest:host stoichiometry). Finally, in order to examine the PN/HP-*β*-CD inclusion complex for which no crystal structure is available, two stable binding models from a docking analysis using AutoDoc Vina [38], both having an 1:1 guest:host stoichiometry but different inclusion modes, were used as the starting structures of MD simulations in case of PN/HP-*β*-CD. The 1:1 stoichiometry for this complex has been shown by Jadhav [39] using Job’s plot analysis and further supported by the A_L_-type profile in the present phase solubility studies. More explicitly, the HP-*β-*CD molecule with degree of substitution (DS) 5 was built using VEGAZZ [40] by removing the methyl groups and arbitrarily adding five 2-hydroxypropyl groups to both rims of DM-*β-*CD. In order to examine the effect of piperine ’s inclusion mode (insertion of the aromatic or piperidine ring in the cavity) in HP-*β-*CD, as the determination of a 3D structure is unattainable, two different models (with high docking scores) representing two different inclusion modes were selected as the starting systems for the PN/HP-*β-*CD case. Charges to 2-hydroxypropyl groups of modified CDs were applied with GAMESS [41]. Consequently, MD simulations were performed for the five complexes by using the Amber12 suite [42]. The q4 md-CD force field [43] was applied to all modified CD atoms, while atoms of native *β-*CD molecules were treated with GLYCAM [44]. The piperine geometry was optimised following the AM1BCC methodology with the program ANTECHAMBER. In all cases, the formed cyclodextrin inclusion complex was initially solvated with TIP3P waters [45] in a periodic, octahedral box forming a 10 Å thick water shell around the structure using xLEaP. Hydrogen atoms were also added with xLEaP in all systems.

MD calculations and minimizations were carried out with SANDER. Periodic boundary conditions were imposed by means of the particle mesh Ewald method using a 10 Å limit for the direct space sum. The protocol included energy minimization for hydrogen atoms with positional restraints of 50 kcal mol^−1^ Å^−2^ on the non-hydrogen atoms, heating equilibration of the solvent in the canonical (NVT) ensemble using positional restraints and the Berendsen thermostat algorithm with coupling constants of 0.5 ps to control temperature and pressure, unrestrained energy minimization, gradual temperature increase from 5 to 300 K with 10 kcal mol^−1^ Å^−2^ restraints on the atoms of the inclusion complex, gradual release of the restraints in successive steps at 300 K and density equilibration in the isobaric-isothermal (NPT) ensemble for 250 ps. Subsequently, production runs using a Berendsen-type algorithm with coupling constants of 1.0 ps were carried out under physiological conditions for additional 12 ns in the NPT ensemble. Root mean square deviation (RMSD) calculations, as well as geometric (H-H bond distance monitoring) analysis of the examined systems were performed by the CPPTRAJ module [46] of Amber12 and VMD [47].

The well-known post-processing single-average MM/GBSA methodology [48] implemented in AMBER [49] was used for investigating the binding free energy of PN to CD hosts in solution. In the MM/GBSA approach, the free energy Δ*G*_binding_ = *G*_complex_ − *G*_host_ − *G*_guest_ for the binding of the guest to the host to form the complex, can be expressed as [50]:

Δ*G*_binding_ = Δ*H* − *T* Δ*S =* Δ*E*_MM_ + Δ*G*_solvation_ − *T* Δ*S* (1)

where Δ*E*_MM_, Δ*G*_solvation_ and −*T* Δ*S* are the changes in the gas-phase molecular mechanics (MM) energy, solvation free energy, and conformational entropy upon PN encapsulation in the host cavity. The Δ*E*_MM_ is achieved from the combination of the electrostatic (Δ*E*_ele_) and van der Waals (Δ*E*_vdW_) energies, whereas the Δ*G*_solvation_ is calculated using Equation (2),

Δ*G*_solvation_ = Δ*G*_GB_ + Δ*G*_nonpolar_ (2)

where Δ*G*_GB_ is the electrostatic solvation energy (polar contribution) calculated using the GB model and Δ*G*_nonpolar_ is the nonpolar contribution between the solute and the continuum solvent, estimated using the solvent-accessible surface area (SASA) [51,52]:

Δ*G*_nonpolar_ = *γ*·SASA + *b* (3)

The change in entropy –*T* Δ*S* is obtained using the NMODE module of AMBER [49]. However, the estimation of the entropy term is often problematic as the normal mode lacks information of the conformational entropy and alternative methods do not give converged results [48]. Thus, although calculated, this term is usually neglected in the comparison between binding affinities of similar inclusion complexes.

## 3. Results

### 3.1. Phase Solubility Analysis

The detection wavelength for the calibration was set at 345 nm. The calibration graph was linear in the range of 0.00125–0.02 mM for the PN. The regression equation of the curve and the coefficient of determination (*R*^2^) were y = 39.19x+0.0009 and *R*^2^ = 0.99 (Supplementary Figure S1). Measurements were performed in triplicate.

**Figure 2:**
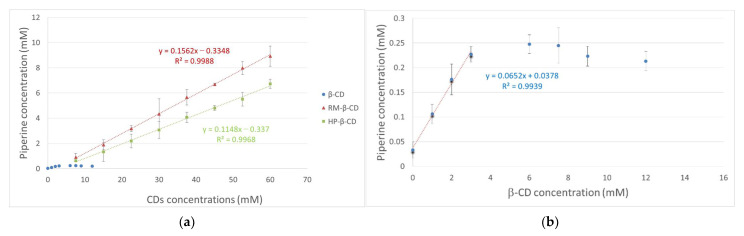
(**a**) Phase solubility diagrams of PN inclusion complexes with *β*-CD (in the range of 0–16 mM), RM-*β*-CD and HP-*β*-CD (0–60 mM) in water media at 25 °C; (**b**) PN increases linearly with increasing *β*-CD only in the range of 1–3 mM. (*n* = 3), (●) *β*-CD, (▲) RM-*β*-CD, (■) HP-*β*-CD.

The phase solubility diagram of PN as a function of various concentrations of *β*-CD and its derivatives (RM-*β*-CD and HP-*β*-CD) at 298 K is shown in Figure 2. The solubility of PN increases linearly with increasing *β*-CD concentration only in the range of 1–3 mM indicating a typical B_s_-type system. On the contrary, both phase solubility diagrams observed for PN versus RM-*β*-CD and HP-*β*-CD hosts respectively, show typical A_L_-type systems suggesting formation inclusion complexes with a 1:1 host:guest stoichiometry. The binding constant (*K_c_*) and the complexation efficiency (CE) values of the inclusion complexes for the two latter cases as well as for the linear portion of the PN/*β*-CD graph are derived from the respective diagrams using the following equations:

*K_c_* = slope/[*S*_0_(1 − slope)], (4)

CE = slope/(1 − slope),(5)

where *S*_0_ is the intercept.

The results from the above experiments are summarized in Table 2. Poorly soluble drugs (with intrinsic solubility values below about 0.1 mM) usually show negative intercept deviation, *S_int_* < *S*_0_ (*S_int_*, intrinsic and *S*_0_ solubility determined in pure water), resulting in *A_L_*- type profiles in pure aqueous solutions [25]. Piperine falls into this category as its intrinsic solubility in water (*S*_int_) has been measured at 0.0229 mM by Ezawa et al. [19], whereas in the present work it was determined as *S*_int_ = 0.0378 mM. The phase-solubility profiles of PN/RM-*β*-CD and PN/HP-*β*-CD (Figure 2) are of *A_L_*- type, giving negative *S_int_* values. In order to estimate the apparent *K*_c_ values for these complexes, the determined *S*_0_ value was used in Equation (4) instead of the intrinsic *S_ini_*. On the other hand, the numerical value of CE calculated by equation (5), expressing the concentration ratio between cyclodextrin in a complex and free cyclodextrin, is only dependent on the slope of the phase solubility profile and thus more reliable than the estimated *K*_c_ value [53].

According to the calculated CE values, the rank order of the PN complexation efficiency with the three examined hosts is: RM-*β*-CD >HP-*β*-CD > *β*-CD. Although the determination of *K*_C_ by the phase-solubility profiles is significantly affected by the low PN solubility, the same rank order is also observed according to these values (Table 2). However, it is more secure to compare between only the PN inclusion complexes in RM-*β*-CD and HP-*β*-CD, both presenting an A_L_-type profile, where a stronger binding affinity is observed in RM-*β*-CD than HP-*β*-CD. In the case of the PN/*β*-CD phase-solubility profile (Figure 2), the solubility of PN increases linearly with increasing *β*-CD only in the range of 1–3 mM, indicating a typical B_s_-type profile. The *K_C_* value in this case is estimated by the linear portion of the profile (Table 2). This profile type is not unusual for inclusion compounds of drugs in native *β*-CDs as the solubility limit of the drug/*β*-CD complex is reached within the concentration range of the *β*-CD. A B-type profile for PN/*β*-CD, with the same maximum solubility for piperine at 3 mM of *β*-CD, has also been observed previously by Ezawa et al. [19]. 

### 3.2. The Crystal Structure of PN/β-CD

**Figure 3:**
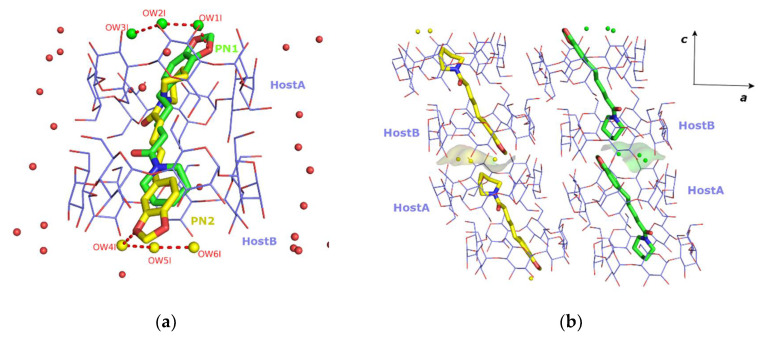

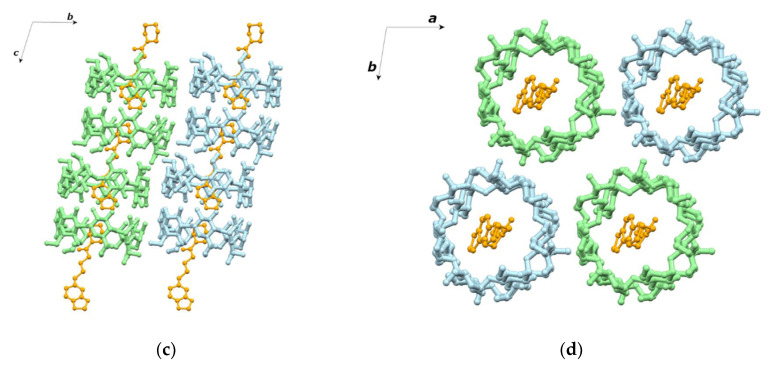
(**a**) The asymmetric unit of PN/*β-*CD inclusion complex. A piperine (PN) molecule, disordered over two sites (PN1 green and PN2 yellow) of opposite orientation, is encapsulated in the cavities of two successive *β-*CDs (HostA and HostB) arranged in a tail-to-tail mode. (**b**) three water molecules, tethered via hydrogen bonds to an oxygen atom of the PN’s benzodioxole group, fill the void within the head-to-head *β*-CD dimers formed as usual via intermolecular hydrogen bonds between the secondary hydroxyls of *β-*CDs. (**c**,**d**) The crystal packing is characterized as channel mode (*CH*) along *c* axis. Only the PN2 site is depicted and hydrogens and waters are omitted for clarity. Images were generated by PyMoL and Mercury.

The crystallographic data of the PN/*β*-CD inclusion complex are summarized in Table 1. PN/*β*-CD crystallizes in the triclinic space group (*P*1) with a 1:2 guest:host stoichiometry, as two *β*-CD host molecules (denoted as hostA and hostB) encapsulate one piperine molecule. The two *β*-CD molecules hosting the piperine molecule, are arranged so that the narrow rim (tail) of the one faces the narrow rim of the other (tail-to-tail mode). The guest molecule is found accommodated “axially” in the hosts’ cavity, occupying two sites (site PN1 and site PN2 with 0.5 occupancy each) of opposite orientation and stabilized by forming numerous van der Waals and C-H…O interactions with the inner cavity of the hosts. In both occupied sites a small network of three water molecules is tethered to an oxygen atom of the guest’s benzodioxole group via hydrogen bonds (Figure 3a). These water molecules fill the void within the head-to-head *β*-CD dimers, formed via the usually observed intermolecular hydrogen bonds between the O3*n*-H hydroxyl groups of the adjacent *β*-CD molecules (Figure 3b). Ezawa et al. [18,20], by using DSC and PXRD methods, had predicted the existence of water molecules inside the *β*-CD cavities for the particular inclusion complex as well as the 1:2 guest:host stoichiometry. The *β*-CD dimers stack along the crystallographic *c*-axis, the angle between their approximate seven-fold axis and *c*-axis being 9.8°, and form layers along the *ab* crystal plane. The shift between two successive dimers along the *c*-axis is 3.1 Å. This displacement is within the range of 2.7 to 3.1 Å observed in the cases of the dimeric structures crystallizing according to the channel (*CH*) packing mode (Figure 3c,d).

Moreover, 18.5 additional water molecules occupying 20 sites have been defined in the unit cell bridging via the hydrogen bonds adjacent complex units within the same and neighbouring channels. The observed host–guest interactions along with the extended hydrogen bond network between water molecules, hosts and guest are listed analytically in Supplementary Table S1.

The conformation of the two hosts is described analytically by listing some geometrical features of the molecules in Supplementary Table S2. The glucosidic O4*n* atoms in both hosts form nearly regular heptagons, which are essentially planar, as indicated by their distances from their approximate centroids (*d*_K_), the distance between adjacent O4*n* atoms (*d*) and their deviations (*dev*) from the O4*n* mean plane. The glycosidic residues (G*_n_*, *n* = 1, …, 7) have positive tilt angles, indicating that their primary sides incline towards the approximate sevenfold axis of the cavity. The majority of hydroxyl groups in both hostA and hostB have the *gauche-gauche* conformation pointing outwards from the cavity. Two hydroxyl groups in hostA (residues 3 and 5) illustrate *gauche-trans* conformations pointing into the cavity, while G1 and G7 units adopt both *gg* and *gt* conformations. In host B, only G2 and G4 residues adopt both *gg* and *gt* conformations.

A Cambridge Structural Database (CSD) search query resulted in two entries with comparable unit cells: the crystal structure of the inclusion complex of *β*-CD with (-)-linallol (CCDC code: UKIXAP) [54] and that of the inclusion complex of *β*-CD with fentanyl (CCDC code: SATFEB) [55], sharing 96% and 91% unit cell resemblance with the PN/*β-*CD, respectively. In the SATFEB entry, the guest:host stoichiometry is also 1:2 and the guest is accommodated inside two successive hosts, similar to piperine.

### 3.3. The Crystal Structure of PN/DM-β-CD

**Figure 4:**
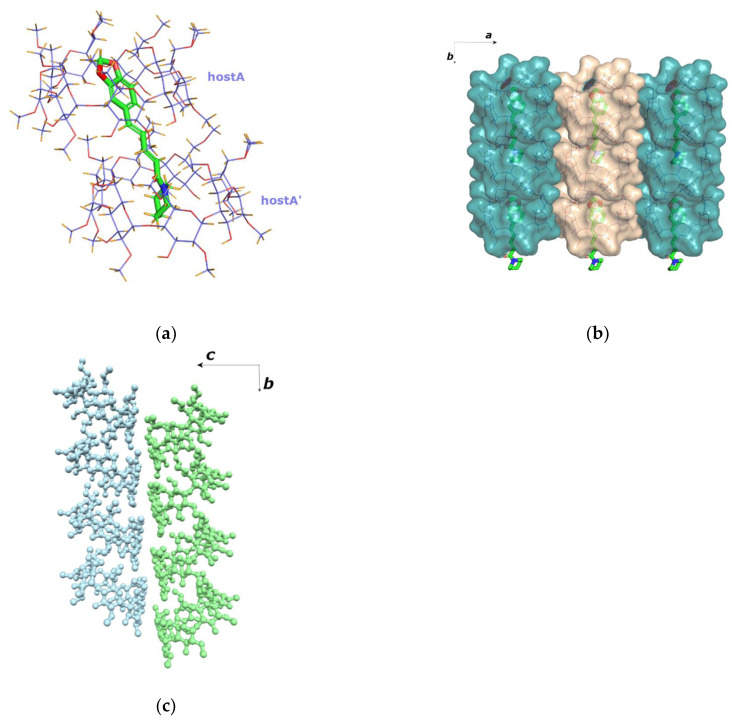
(**a**) A piperine molecule is encapsulated in the hydrophobic moiety formed by two symmetry related DM-*β-*CD hosts. (**b**,**c**) The complex units form columns along the *b*-axis which are stacked parallel.

The PN/DM-*β*-CD complex crystallizes in the space group *P*2_1_ with lattice parameters quoted in Table 1. Its asymmetric unit contains one host molecule and one partially entrapped PN guest molecule (with sof 0.5), found with its 1,3-Benzodioxole moiety entering the DM-*β*-CD cavity (hostA) from its secondary (wide) rim. The remaining part of the guest molecule is also encapsulated in the hydrophobic cavity of the symmetry related DM-*β*-CD (hostA’), entering it from its primary (narrow) rim. Thus, a piperine molecule threads two DM-*β*-CD molecules arranged in a head-to-tail mode (shifted by 6.074(18) Å), forming a 1:2 guest:host inclusion complex in the crystalline state (Figure 4a). These complex units are arranged in columns along the crystallographic axis-*b*, which are tightly packed parallel (based on the orientation of individual DM-*β-*CD units) via numerous C–H···O and H···H closed shell interactions between host molecules, without the aim of any water molecule (Figure 4b,c). No hydrogen bonds between guest and DM-*β*-CD or neighbouring host are observed (Supplementary Table S1).

The pyranose residues of the DM-*β*-CD host molecule have the usual ^4^*C*_1_ conformation and their geometric features are reported in Supplementary Table S3. The values in this table indicate that the host DM-*β-*CD molecule retains in general its torus-like macrocycle shape and the round conformation. This is due to the formation of the well-known intramolecular interglucose O3(*n*)-H···O2(*n* + 1) hydrogen bonds. The heptagon formed by the O4*n* glucosidic atoms of the host seems to slightly deviate from the ideal planarity and sevenfold symmetry (Supplementary Table S3 and Supplementary Figure S2). Five primary methoxy groups of the DM-*β-*CD molecule have the *gauche-gauche* (*gg*) conformation pointing outwards from the cavity, while one group has the *gauche-trans* (*gt*) conformation pointing inwards and one adopts both conformations (Supplementary Table S3).

Two entries with comparable unit cells (with resemblance of about 96%) were found in a CSD search (YIVXUX and YOJSUL) but none of them is a crystal structure of cyclodextrin inclusion complex. The uniqueness of the investigated crystal packing can be attributed to the lack of bridging water molecules as in the case of a-naphthaleneacetic acid/DM-*β-*CD complex [56].

### 3.4. The Crystal Structure of PN/TM-β-CD

**Figure 5:**
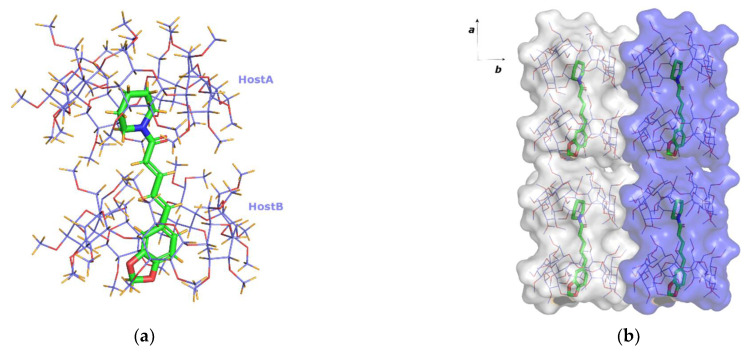

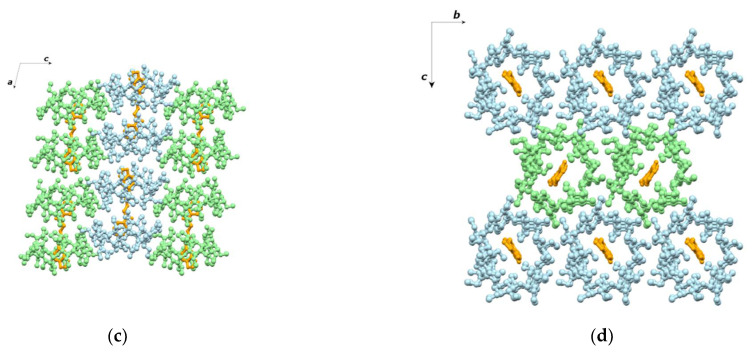
(**a**) In the asymmetric unit, one piperine (PN) molecule (shown as green sticks) is fully encapsulated inside two TM-*β-*CD host molecules (hostA and hostB shown as purple lines). (**b**) The projection of the *ab* plane reveals two parallel columns. (**c**) The projection of the *ac* plane reveals antiparallel columns. (**d**) The crystal packing is characterised by the formation of channels along the *a* axis.

The inclusion complex of PN/TM-*β-*CD also crystallizes in the *P*2_1_ space group. Unit cell parameters and further crystal lattice details are quoted in Table 1. The asymmetric unit of the structure consists of two hosts and one guest molecule (1:2 guest:host stoichiometry). Similar to the PN/DM-*β-*CD crystal structure, the piperine is found encapsulated in the extended hydrophobic moiety formed by two TM-*β-*CD molecules that are arranged in a head-to-tail mode along the crystallographic *a*-axis (Figure 5a). The O4(*n*) planes of the two hosts form an angle of 2.663(2)° with each other and their centroids are shifted by 1.924(7) Å. However, the orientation of the guest in the PN/TM-*β-*CD inclusion complex is opposite to that of the guest in PN/DM-*β-*CD. In particular, the piperidine ring of the guest is accommodated in the hostA cavity (upper CD), entering from its wide rim and forming an angle of 70.81(19)° with its O4(*n*) plane, whereas the 1,3-benzodioxole moiety of the guest is accommodated in the hostB cavity (lower CD), entering from its narrow rim and forming an angle of 78.3(3)° with its O4(*n*) plane. Host–guest interactions include CH..O bonds and multiple H-H interactions (Supplementary Table S1).

In Supplementary Table S4, some geometrical features regarding the conformation of the two TM*-β*-CD molecules are given, the values of which reveal that both host macrocycles are severely distorted due to the absence of any intramolecular interglucose hydrogen bonds. The glycosidic O4(*n*) atoms deviate significantly from their mean plane (their distance from the mean plane ranging from −0.480 to 0.579 Å for HostA and −0.582 to 0.545 Å for HostB). The distances between the O4(*n*) atoms and their centroid also vary significantly (from 4.362 to 5.387 Å for hostA and from 4.378 to 5.450 Å for hostB) indicating two distorted macrocycles that have the shape of elliptical heptagons. The tilt angles of the permethylated glucopyranose units of the host (*τ***:** tilt angles between the optimum O4(*n*) mean plane and the mean plane of the O4(*n*−1), C1(*n*), C4(*n*), and O4(*n*) atoms) span a wide range from −14.64° to 43.63° (hostA) and from −24.50° to 39.33° (hostB), contributing to the narrowing of the primary and the broadening of the secondary rim. In hostA, two glucose residues have positive tilt angles (*τ*_1_) indicating that their primary sides incline towards the approximate sevenfold axis of the macrocycle whereas three methyl-glucose residues (G2, G4 and G6) have negative tilt angles. The remaining two glucose residues (G1 and G7) adopt both *gg* and *gt* conformations. In hostB, five residues have the *gg* conformation and the G2 and G4 units have both (*gg* and *gt*). The high *τ* values of G2, G3 and especially G7 of hostA indicate the formation of the characteristic ‘lid’ in the primary region of the host that is usually observed in TM*-β*-CD inclusion complexes [57], preventing the deep penetration of the guest. On the other hand, G1, G2 and G4 of hostB also show high *τ* values, but the host macrocycle is more “open” as almost all residues of hostB have the gauche-gauche (*gg*) conformation. Thus, it is the combination of the two characteristics that plays a crucial role in the overall host conformation.

In the absence of bridge water molecules, the complex units stack in a head-to-tail mode along the twofold screw *a*-axis with their mean O4 planes slanted at 5.38° (for host A) and 3.40° (for hostB) against the *bc* plane (Figure 5c). The complex units of these columns are interconnected via numerous host-adjacent host CH … O and CH/π interactions. The adjacent deployed columns are antiparallel as complex monomers have opposite orientations (Figure 5c,d).

No unit cell match was found by traversing the CSD database with the aim of finding structures with similar cell dimensions based on differences between Krivy-Gruber reduced cells.

### 3.5. MDs Trajectories Analysis

Atomistic simulations for the inclusion complexes of piperine in *β-*CD, DM-*β-*CD, TM-*β-*CD and HP-*β-*CD, as determined by the SC-XRD experiments or produced from docking analysis were undertaken for almost 12 ns at 300 K in explicit water solvent. 

**Figure 6:**
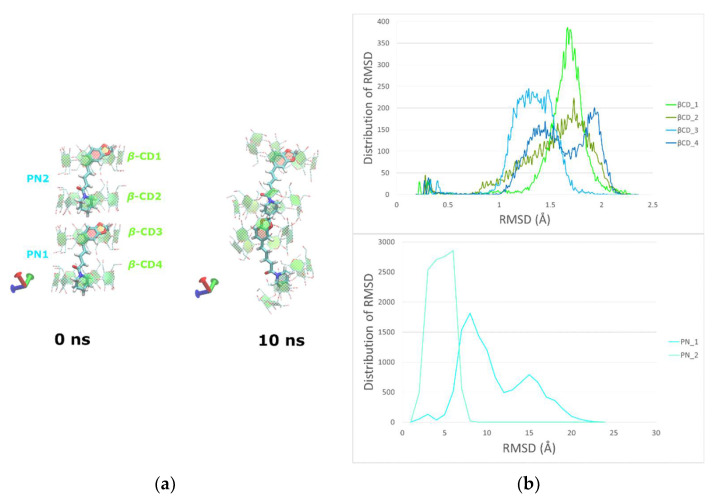
(**a**) Representative snapshots of PN/*β*-CD at 0 and 10 ns. (**b**) The distribution analysis of RMSD values for the four host (*β*-CD1, *β*-CD2, *β*-CD3 and *β*-CD4) and two guest (PN1 and PN2) molecules.

In the case of PN/*β*-CD, an assembly of four consecutive *β*-CDs hosting two piperine molecules was used as the starting model. This choice was made in order to monitor the dynamic behavior of two consecutive tail-to-tail complex units (PN2/*β*-CD1 & *β*-CD2 and PN1/*β*-CD3 & *β*-CD4) as revealed by crystallography, that interlinked via the H-bonds network between the hydroxyls of the *β*-CD’s wide rims that face each other and form the usually observed head-to-head dimer (*β*-CD2–*β*-CD3) (Figure 6a).

By monitoring the frames during the time frame of the simulation, we observed that both PN1 and PN2 guests retain their accommodation in the tail-to-tail host couples with their benzodioxole and piperidine groups always embedded in the *β*-CD cavities. On the other hand, the H-bonds network between the secondary hydroxyls of *β*-CD2 and *β*-CD3 that interconnects the adjacent complex units by a head-to-head mode is disrupted after the 5th ns, causing a severe distortion to the head-to-head facing of these hosts observed in the crystal structure. Although no disassociation of the supramolecular system is observed during the 12 ns time of simulation, probably due to the limited time interval and box size of the simulation, the diagrams of the RMSD values distribution show that the PN1/*β*-CD3 & *β*-CD4 complex unit departs from the crystal arrangement that enforces its head-to-head interlink with the PN2/*β*-CD1 & *β*-CD2 complex unit (Figure 6b and Supplementary Figure S3). The preference of *β*-CD’s dimerization in a head-to-head orientation has been early shown by Bonnet et al. with MM and MD calculations in vacuo [58]. According to these studies *β*-CDs present the head-to-head orientation as the most energetically stable while their number of intermolecular hydrogen bonds (about 8–9) being the largest among all the computed dimers orientations. In the case of PN/*β*-CD, although initially 8 intermolecular H-bonds are observed between the adjacent head faces of the complex units in the starting model based on the crystal structure, this number immediately decreases to about 5 H-bonds, then after the 4th ns it further decreases to about 3 H-bonds and finally after the 10th ns just 2 H-bonds in average are observed (Supplementary Figure S4) indicating that this head-to-head formation is rather unstable.

H-H interactions between host (PN1) and guest (*β*-CD3) are monitored during the simulation. The H14, H17 and H18 atoms of the benzene ring of PN1 interact with the H3 (Supplementary Figure S5) and H5 (Supplementary Figure S6) atoms of *β*-CD3 and these results are in agreement with previous NMR studies of Ezawa et al. [18]. However, the interactions between the aromatic ring’s H atoms and the H6 atoms of the host that the same NMR studies had shown, are not observed here due to the specific inclusion mode, in which the H atoms of the guest are always in close proximity to the wider rim of the host (hydroxyl groups attached to secondary C2 and C3 atoms of the macrocycle) and not with the narrower (C6 atom). The number of contacts between PN1 and PN2 molecules with *β*-CD3-, *β-*CD4 and *β-*CD1-*β-*CD2 duets is illustrated in Supplementary Figure S7**)**.

**Figure 7:**
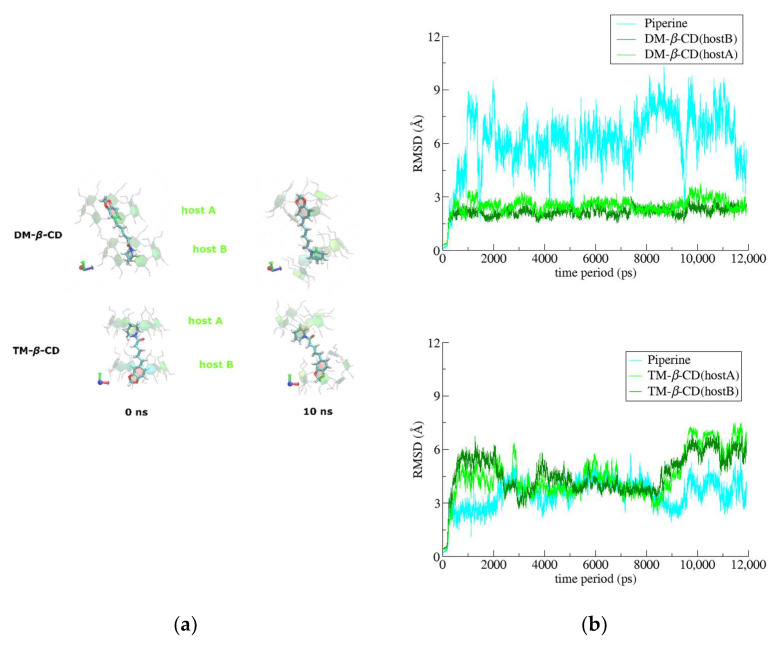
(**a**) Representative snapshots of PN/DM-*β*-CD and PN/TM-*β*-CD inclusion complexes at 0 and 10 ns. (**b**) RMSD to the first frame for the inclusion complexes of PN/DM-*β*-CD (1:2), PN/TM-*β*-CD (1:2) as a function of MD simulations time.

In both cases of PN/DM-*β*-CD and PN/TM-*β*-CD, the piperine adopts an extended conformation threading constantly its two hosts during the time-frame of the simulations (Figure 7a). The depth of immersion of the aromatic rings of the guest in the hosts’ cavities varies significantly during the simulations time, resulting to high RMSD values from the initial, crystallographically determined, pose (Figure 7b). In the case of PN/DM-*β*-CD, the existence of secondary hydroxyls in the wide rim of the hosts enables the formation of intramolecular H-bonds that limits the conformational variations of their cavities. The guest PN is able to move more freely in this relatively rigid hydrophobic environment always tethered by alternate H-bonds between its oxygen atoms and the hosts’ secondary hydroxyls. On the other hand, in the case of the TM-β-CD hosts, the lack of any intra- and inter-molecular hydrogen bond causes a higher flexibility and mobility of these molecules, as it is shown in the RMSD diagram of Figure 7b, whereas the mobility of the encapsulated guest is rather limited, following a pronounced induced-fit mechanism [59].

**Figure 8:**
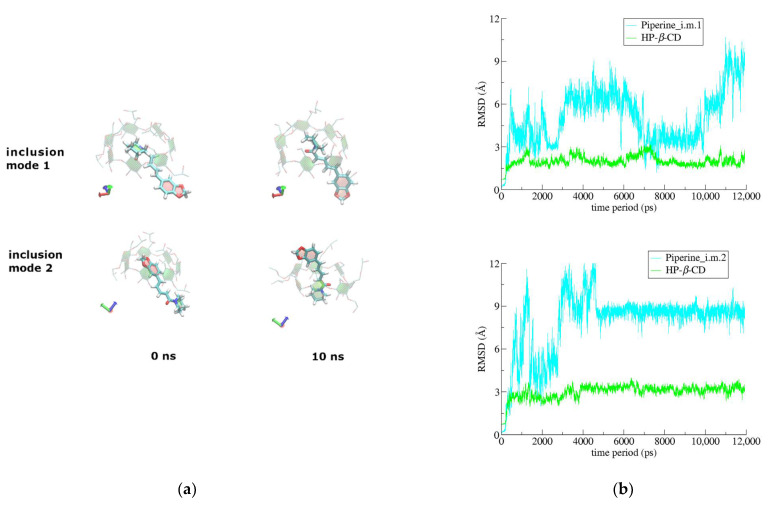
(**a**) Representative snapshots for the two different starting poses of PN/HP-*β*-CD inclusion complex at 0 and 10 ns during simulation time. (**b**) RMSD to the first frame for the inclusion complexes of PN_i.m.1/HP-*β*-CD and PN_i.m.2/HP-*β*-CD as a function of MD simulations time.

Finally, in the case of the PN/HP-*β*-CD complex, where a 1:1 guest:host stoichiometry was chosen, the guest is highly mobile (Figure 8a,b) and the complex is significantly less stable as the binding affinity estimations using the MM/GBSA method indicate (see below). Two inclusion modes were examined: PN-i.m.1 and PN-i.m.2, with the piperidine ring and the 1,3-benzodioxole group of the guest initially encapsulated in the CD cavity, respectively. In both inclusion modes the remaining part of the guest protrudes from the host’s wide rim. By monitoring the frames during the time interval of the simulations, a tendency of the 1,3-benzodioxole group to be exposed to the solvent, forming hydrogen bonds with the water molecules, is observed. In the case of PN-i.m.1, where from the beginning of the simulation the 1,3-benzodioxole group is exposed to the solvent, the guest is highly mobile, whereas in the case of PN-i.m.2, the initially encapsulated 1,3-benzodioxole group escapes from the narrow rim of the host after the 5th ns of the simulation and the piperidine group takes its place in the CD cavity, stabilizing the guest, as it can be seen in the RMSD diagrams (Figure 8a,b). Thus, in both cases the hydrophobic CD cavity is found hosting the piperidine group of the guest and no host-guest disassociation is observed during the time-frame of the simulations (Figure 8a,b). 

Binding affinities for the five examined supramolecular complexes were calculated by the molecular mechanics/generalized Born surface area (MM/GBSA) method as listed in Table 3**.** As expected, in the case of PN/HP-*β-*CD, where a 1:1 guest:host stoichiometry was chosen as indicated by the solubility profile of the complex, the averaged change of van der Waals energies (Δ*E*_vdW_) upon PN inclusion in the host, is about half for both examined inclusion modes compared to the PN inclusion complexes in *β*-CD DM-*β*-CD and TM-*β*-CD where the 1:2 guest:host starting models indicated by the crystallographic studies, were used. The lack of hydroxyls in the case of the permethylated host (TM-*β*-CD) minimizes the formation of guest–host intermolecular H-bonds, thus lowering significantly the (Δ*E*_ele_) term. The existence and lifetime indicative of such H-bonds in the case of the PN/*β-*CD complex is shown in the Supplementary Figure S8 and Table S5.

The estimated binding affinities for the first three cases (PN in *β*-CD, DM-*β*-CD and TM-*β*-CD) that share a common stoichiometry, show quite stable inclusion complexes. A higher value for PN/DM-*β*-CD is observed indicating probably a more stable inclusion complex, although the differences between the calculated Δ*G*_bind_ values fall within the error margins. On the other hand, the estimated binding affinity for both examined inclusion modes of the PN/HP-*β-*CD complex, indicates an unstable complex, although no host–guest disassociation was observed by monitoring the trajectories during the time frame of the simulations. This contradiction could be attributed to the limited time interval of the simulations (12 ns), however, as it is mentioned in Section 2.5, the calculation of the entropic term (*T*·Δ*S*) based on normal mode analysis is often problematic and thus its combination with the relatively low absolute value of the enthalpic term (Δ*H)* due to the 1:1 host:guest starting model, that resulted to the positive Δ*G*_bind_ values for these cases, is quite disputable.

## 4. Discussion

X-ray crystallography studies of the PN inclusion complexes in *β-*CD and its methylated derivatives heptakis(2,6-di-*O*-methyl)-*β*-Cyclodextrin (DM-*β-*CD) and heptakis(2,3,6-tri-*O*-methyl)-*β*-Cyclodextrin (TM-*β-*CD), revealed the formation of inclusion complexes with 1:2 guest:host stoichiometry in the crystalline state. In all determined crystal structures, a piperine molecule is found encapsulated in an extended hydrophobic cavity formed by two hosts which are arranged in a tail-to-tail mode (narrow rim facing the narrow rim) in the case of PN/*β-*CD and in a head-to-tail mode (wide rim facing the narrow rim) in the cases of PN/DM-*β-*CD and PN/TM-*β-*CD. Interestingly, in all cases it is the guest PN molecule that mainly interconnects the two host CDs, threading their cavities. Native *β-*CD hosts usually tend to form head-to-head dimers via a network of intermolecular H-bonds between their secondary hydroxyls. The formation of these dimers is observed in the crystalline state of PN/*β-*CD complexes, however this dimeric cavity is filled by water molecules tethered to the protruding part of an encapsulated PN in the adjacent tail-to-tail *β-*CD couple. This inclusion mode, revealed by the determined crystal structure of the PN/*β-*CD complex, is consistent and further explains previous findings for the PN/*β-*CD complex by DSC and ^1^H-NMR studies. In particular, the existence of water molecules in the *β-*CD cavity have been indicated by previous DSC studies of the PN/*β-*CD complex [18], whereas the interactions between the PN aromatic ring and the H3 and H5 of the *β-*CD that have been previously reported by ^1^H-NMR studies [19] were also observed by monitoring the host-guest H-H proximity during the MD simulations performed in this work for the PN/*β-*CD complex in an aqueous environment. The H-bonds network, which forms the above mentioned head-to-head *β-*CD dimers, that interconnect two adjacent tail-to-tail complex units, is not maintained during the time-frame of the MD simulations, although the inclusion complexes remain stable. On the other hand, in the cases of the DM-*β-*CD and TM-*β-*CD hosts, this kind of head-to-head dimers cannot be formed due to the limitation or complete absence of hydroxyls in their rims. Thus, the couple of hosts in the corresponding complex units is arranged in a head-to-tail mode forming columns in the crystalline state.

The stability of the inclusion modes revealed by X-ray crystallography was examined in an explicit water environment and in the absence of crystal contacts by MD studies. All inclusion complexes remain stable during the 12 ns simulations and MM/GBSA calculations showed the high binding affinity of PN for the *β-*CD, DM-*β-*CD and TM-*β-*CD hosts in their 1:2 guest:host complexes. On the other hand, in MD simulations performed for the PN/HP-*β*-CD inclusion complex by using docked starting models of 1:1 guest:host stoichiometry, as indicated by the solubility profile of the complex, although no complex dissociation was observed, the estimated binding affinities were significantly lower. This result, although expected due to the 1:1 guest:host stoichiometry of the complex, is not consistent with the rank order of the PN affinity with *β*-CD, RM-*β*-CD and HP-*β*-CD hosts (RM-*β*-CD > HP-*β*-CD > *β*-CD), according to the estimated *Kc* and CE values by phase-solubility studies. The solubility profiles for these complexes indicate a B_S_-type for PN/*β*-CD and an A_L_-type for PN/RM-*β*-CD and PN/HP-*β*-CD, thus the CE and *Kc* calculations were made for 1:1 guest:host complexes. 

Ezawa et al. [18] have indicated a 1:1 guest:host stoichiometry for PN/*β-*CD in solution by a Job’s plot. Moreover, in a following work of Ezawa et al. [19] a B-type solubility profile, similar to the one observed in this work, and a significant higher estimation binding constant of 3244 M^−1^ was reported for PN/*β-*CD. However, in a recent work by Alshehri et al. [60] a binding constant of just 287 M^−1^, estimated by an A-type solubility profile of PN/*β-*CD, was reported. This low value and the A-type profile is disputable for the following reason: It is well documented that CD inclusion complexes, especially those of the natural CDs, have tendency to self-assemble in aqueous solutions to form aggregates, giving rise to characteristic B-type phase-solubility diagrams [61]. The self-aggregation increases with increasing cyclodextrin concentration. The increase of *β-*CD concentration in the phase solubility study by Alshehri et al. is not high enough to display the B-curve plateau. By comparing the binding constant of 3244 M^−1^ estimated by Ezawa et al. [19] to the one presented in this work (1800 ± 300) M^−1^, we should note that *Kc* as well as CE values for the B-type complexes are estimated as 1:1 guest:host complexes by the limited linear portion of the profile. Moreover, the *K_C_* value determined by Equation (4) is strongly affected by the *S*_0_ value which is usually very inaccurate for compounds with *S*_0_ < 0.1 mg/mL [62] like piperine. All these may cause the difference observed between these estimations. In addition, the theoretical calculations of this work, based on the 1:2 guest:host complex revealed by crystallography in aqueous solution, resulted in a high binding affinity for this complex. These results can justify the high *K_c_* value estimated by Ezawa et al. [19] considering that the stability constants obtained from phase-solubility diagrams are estimations for 1:1 complexes but are the most frequently observed constants that are composed of a number of true stability constants for multiple types of coexisting water-soluble drug complexes in the aqueous complexation media. Moreover, it is well documented that the self-aggregation that causes a B-type solubility profile, as the one observed for this complex, is usually observed for multi-component ternary and quaternary CD complexes [63]. Thus, according to our theoretical calculations, the overall *Kc* obtained by the phase solubility diagrams, is expected to be high (at least higher than that of PN/HP-*β*-CD) as estimated by Ezawa et al. [19].

Moreover, in crystalline state, it is observed that the formation of *β*-CD channels hosting PN molecules bridged by water molecules which are entrapped in the head-to-head *β*-CD interfaces. Although this structure resembles the “molecular necklace” of a linear CD pseudopolyrotaxane obtained in the solid state [64], the inclusion compounds of the linear assembly threaded by the CD rings are not directly interconnected, resulting in an unstable “necklace” in solution. Theoretical studies by Anconi et al. [65] have shown that CD pair interaction plays a major role in the probability distribution of the entities formed in the self-assembly system of polymers threaded by CDs. Although in this case no polymer is threaded in the CDs’ nanotube, it is interesting to notice the following: A tail-to-tail inclusion mode, as revealed by the crystal structures of PN/*β*-CD presented in this work, and other similar structures that will be presented in forthcoming works (e.g., the inclusion complex of capsaicin in *β*-CD; unpublished data), results in a B_s_-type solubility profile, meaning a low solubility of the complex caused by self-aggregation of the complex units. On the other hand, a 1:2 guest:(head-to-head) host inclusion mode, as observed in the crystal structure of cholesterol in *β*-CD [66] is correlated with an A-type solubility profile [67], indicating that the complex units do not aggregate so intensively. Thus, the inclusion mode in the complex units of the 1:2 guest:host *β*-CD dimers, in the sense of whether the wide or the narrow rims are the open ends interconnecting adjacent complex units, plays a crucial role in the self-aggregation and thus solubility of the inclusion complex. However, this topic has to be further investigated.

For all the above reasons, it is safer to compare the *K_C_* and CE values estimated by phase solubility studies solely between the PN/RM-*β*-CD and PN/HP-*β*-CD inclusion complexes, that both present an A_L_-type solubility profile. From this comparison, a stronger binding affinity is indicated for PN in RM-*β*-CD than HP-*β*-CD. The low binding constant of PN/HP-*β*-CD in solution has also been reported by Imam et al. [68] estimating a value of 238 M^−1^ by phase solubility studies. Thus, in agreement with our MM/GBSA calculations, PN has lower affinity for HP-*β*-CD than methylated *β*-CDs.

## 5. Conclusions

The determination of the crystal structure of the PN inclusion complexes in *β-*CD, DM-*β-*CD and TM-*β-*CD presented in this work, gives unique and valuable information about the stoichiometry and geometry of the complexes that conclusively clarifies the inclusion mode of piperine in these host molecules. In all determined structures, inclusion complexes of 1:2 guest:host stoichiometry were found in the crystalline state. The guest PN molecule threads the hydrophobic cavities of the hosts which are arranged as couples in a tail-to-tail mode in the case of PN/*β-*CD and in a head-to-tail mode in the cases of PN/DM-*β-*CD and PN/TM-*β-*CD.

Complement MD studies were performed for the crystallographically determined structures in order to monitor the dynamic behavior and the stability of the complexes in an aqueous environment and in the absence of crystal contacts. All inclusion complexes remain stable during the 12 ns simulations and MM/GBSA calculations showed the high binding affinity of PN for the *β-*CD, DM-*β-*CD and TM-*β-*CD hosts. Moreover, MD simulations were performed for the PN/HP-*β-*CD inclusion complex by using docked starting models of 1:1 guest:host stoichiometry, as indicated by the solubility profile of the complex. By monitoring the trajectories during the time-frame of the simulations the complex also remains stable although the absolute values of the estimated binding affinities (neglecting the entropic term) were significantly lower than those of the other examined complexes.

Finally, phase solubility studies were carried out for the PN/*β*-CD, PN/RM-*β-*CD and PN/HP-*β-*CD inclusion complexes in order to examine the solubility profile and estimate the apparent stability constant (K_1:1_) and the complexation efficiency (CE) for these complexes in aqueous solution. The low solubility of PN/*β-*CD resulted in a B_S_ solubility profile and the estimation of the K_1:1_ and CE values, under the assumption of a 1:1 guest:host complex, is quite disputable. Although the complex units in solution may differ from those in crystalline state, the overall 1:1 guest:host stoichiometry used in these estimations, resulted to low values relative to the other complexes. This is inconsistent with the high binding affinity values given by MM/GBSA end-state energy calculations that were based on the MD simulations of the 1:2 guest:host crystal structure in an aqueous environment. Thus, it is safer to compare the estimated K_1:1_ and CE values only between the PN/RM-*β*-CD and PN/HP-*β*-CD complexes, both of A_L_-type profile, which show a higher binding affinity for the former than the latter complex in accordance with the calculated values using the MM/GBSA method.

## Figures and Tables

**Table 1:** Experimental details, crystal data and refinement statistics.

	PN/*β*-CD	PN/DM-*β*-CD	PN/TM-*β*-CD
Crystal data			
Chemical formula	2(C_42_H_70_O_35_)C_17_H_19_NO_3_· 21.5(H_2_O)	C_56_H_98_O_35_·0.5(C_17_H_19_NO_3_)	2(C_63_H_112_O_35_)C_17_H_19_NO_3_
Mr	2902.32	1474.00	3141.35
Crystal system, space group	Triclinic, *P*1	Monoclinic, *P*2_1_	Monoclinic, *P*2_1_
Temperature (K)	100	120	100
*a*, *b*, *c* (Å)	15.435 (2), 15.452 (2), 15.502 (2)	15.364 (4), 10.3253 (16), 25.006 (5)	20.8000 (14), 14.7391 (10), 27.0968 (18)
*β* (°)	104.761 (5), 100.770 (5), 104.207 (6)	106.552 (16)	96.214 (4)
V (Å^3^)	3341.9 (6)	3802.4 (13)	8258.3 (10)
Z	1	2	2
Radiation type	Cu *Ka*	Cu *Ka*	Cu *Ka*
μ (mm^−1^)	1.14	0.90	0.86
Crystal size (mm^3^)	0.5 × 0.4 × 0.2	0.8 × 0.12 × 0.1	0.22 × 0.16 × 0.08
Data collection			
Diffractometer	Bruker *APEX*-II	Bruker *APEX*-II	Bruker *APEX*-II
Absorption correction	Multi-scan *TWINABS—*Bruker AXS scaling for twinned crystals—Version 2012/1	Multi-scan *SADABS2016*/2—Bruker AXS area detector scaling and absorption correction	Multi-scan *SADABS2016*/2—Bruker AXS area detector scaling and absorption correction
T_min_, T_max_	0.607, 0.796	0.352, 0.752	0.582, 0.75
No. of measured, independent and observed [I > 2σ(I)] reflections	16,593 (domain 1) 17,055 (domain 2) 17,875 (composites) 21,234, 21,234, 20,381	58,942, 12,341, 10,665	88,314, 13,469, 11,219
R_int_	0.05	0.072	0.089
(sin θ/λ)_max_ (Å^−1^)	0.581	0.582	0.465
Refinement			
*R*[*F*^2^ > 2σ(*F*^2^)], *wR*(*F*^2^), *S*	0.076, 0.203, 1.02	0.100, 0.271, 1.06	0.118, 0.316, 1.04
No. of reflections	21,234	12,341	13,469
No. of parameters	1688	1044	1224
No. of restraints	209	77	213
H-atom treatment	H-atom parameters constrained	H-atom parameters constrained	H-atom parameters constrained
Δρ_max_, Δρ_min_ (e Å^−3^)	0.74, −0.48	0.48, −0.45	0.67, −0.55

**Table 2:** Stability constants *K_c_* in (M^−1^) and complexation efficiency CE (%) values of PN inclusion complexes with native and two modified *β*-CD derivatives at 25 °C (*n* = 3).

Complexes	Type	Linear Equation	*R* ^2^	*K_c_* (M^−1^)	CE (%)
PN/*β*-CD	B_s_	y = 0.0652x + 0.0378 (for linear portion)	0.9939	1800 ± 300	7.0 ± 0.4
PN/RM-*β*-CD	A_L_-	y = 0.1562x − 0.3348	0.9988	4900 ± 500	18.5 ± 0.3
PN/HP-*β*-CD	A_L_-	y = 0.1148x − 0.3337	0.9968	3400 ± 300	13.0 ± 0.3

**Table 3:** Binding free energies and their standard deviations (kcal/mole) resulting from MM/GBSA analysis of the inclusion compounds of PN in *β-*CD, DM-*β-*CD, TM-*β-*CD and HP-*β-*CD with guest:host ratios of 2:4, 1:2, 1:2 and 1:1 (two inclusion modes), respectively.

	PN1/ *β-*CD	PN2/ *β*-CD	PN/ DM-*β-*CD	PN/ TM-*β-*CD	PN_i.m.1/ HP-*β-*CD	PN_i.m.2/ HP-*β-*CD
Δ*E*_vdW_	−51.70 ± 4.08	−50.79 ± 4.27	−51.16 ± 3.01	−53.41 ± 3.60	−27.15 ± 2.24	−26.23 ± 2.60
Δ*E*_ele_	−10.41 ± 4.64	−9.66 ± 4.18	−11.09 ± 4.08	−6.43 ± 2.58	−5.79 ± 3.15	−7.00 ± 3.16
Δ*E*_MM_^a^	−62.11 ± 6.25	−60.45 ± 6.45	−62.25 ± 5.36	−59.84 ± 4.84	−32.94 ± 3.86	−33.27 ± 3.90
Δ*G*_GB_	26.68 ± 3.91	25.31 ± 4.01	26.43 ± 3.84	27.16 ± 3.00	23.64 ± 3.68	25.43 ± 3.28
Δ*G*_nonpolar_	−5.44 ± 0.30	−5.33 ± 0.25	−5.49 ± 0.20	−5.37 ± 0.23	−3.28 ± 0.25	−3.29 ± 0.34
Δ*G*_solvation_^b^	21.24 ± 3.84	19.99 ± 3.90	20.93 ± 3.83	21.78 ± 2.93	20.35 ± 3.59	22.16 ± 3.12
Δ*H* ^c^	−40.87 ± 4.60	−40.47 ± 4.56	−41.31 ± 2.94	−38.06 ± 3.20	−12.58 ± 2.08	−11.11 ± 2.29
T·Δ*S* ^d^	−20.43 ± 3.76	−19.93 ± 3.14	−16.51 ± 4.16	−17.61 ± 3.78	−15.63 ± 1.48	−16.31 ± 1.71
Δ*G*_binding_^e^	−20.43 ± 5.94	−20.53 ± 5.53	−24.80 ± 5.10	−20.45 ± 4.95	+3.04 ± 2.69	+5.20 ± 2.86

Δ*E*_vdW_ = van der Waals contribution from molecular mechanics; Δ*E*_ele_ = electrostatic energy as calculated by the molecular mechanics force field; Δ*G*_GB_ = the electrostatic solvation energy (polar contribution) calculated using the GB model; Δ*G*_nonpolar_ = nonpolar contribution to the solvation free energy, calculated by the solvent-accessible surface area (SASA) method; ^a^ Δ*E*_MM_ = Δ*E*_vdW_ + Δ*E*_ele_; ^b^ Δ*G*_solvation_ = Δ*G*_GB_ + Δ*G*_nonpolar_; ^c^ Δ*H* = Δ*G*_solvation_ + Δ*E*_MM;_^d^ *T* ΔS entropic term calculated by normal mode analysis; ^e^ Δ*G*_binding_ = Δ*H* − *T*·Δ*S.*

## Data Availability

Crystallographic data have been deposited into the Cambridge Structural Database (CSD) under the deposition numbers CCDC: 2063126, 2022888 and 2063127. Further data presented in this study are available on request from the corresponding author.

## Supplementary Materials

The following supporting information can be downloaded at: https://www.mdpi.com/article/10.3390/biom12121762/s1: Figure S1. The solubility profile of pure Piperine in an equimolar water: methanol solution (n = 3). Figure S2. The distances and angles as defined in Tables S2–S4. In particular the following are presented: (**a**) *d* = O4*n*…O4(*n +* 1) distances; (**b**) *d_K_* = distances of the approximate center K of the O4*n* heptagon from the O4*n* atoms; (**c**) *dev* = deviations of the O4*n* atoms from their least-squares plane; (**d**) *φ* =O4(*n*−1)…O4*n* … O4(*n +* 1) angles; (**e**) *φ_K_* = O4*n*… K…O4(*n* + 1) angles. All distances are given in Å and angles in (°). Figure S3. RMSD to first frame vs. time plots for the four host and two guest molecules in the supramolecular ensemble of PN/*β*-CD. Figure S4. Number of H-bonds between hydroxyls of the wide rims of adjacent PN/*β*-CD complex units within the same channel. Figure S5. H-H close contacts between guest’s (PN1) H14, H17 and H18 atoms and host’s (*β*-CD3) H3 atoms of specific glucose residues (G1, G2, G3, G4, G5 and G6). Figure S6. H-H close contacts between guest’s (PN1) H14, H17 and H18 atoms and host’s (*β*-CD3) H5 atoms of residues G1, G2, G3, G4, G5 and G6. Figure S7. Plots of contacts between PN1 and PN2 guests and β-CD3/β-CD4 and β-CD1/β-CD2 host duets, respectively in PN/β-CD complex case. Figure S8. Number of H-bonds between hosts and guests in PN/*β*-CD complex case. Table S1. Main intramolecular interactions present in the crystal structures of PN/*β*-CD, PN/DM-*β*-CD and PN/TM-*β*-CD inclusion complexes. Table S2. Conformational characteristics of the two *β*-CD host molecules in the PN/*β*-CD structure. Table S3. Conformational characteristics of the DM-*β*-CD host molecule in the PN/DM-*β*-CD structure. Table S4. Conformational characteristics of the two TM-*β*-CD host molecules in the PN/TM-*β*-CD structure. Table S5. H bond analysis for the two encapsulated PN molecules (PN_1 and PN_2) in their complex with four *β*-CDs during the 12ns MD simulation.

## References

[1] Butt M.S., Pasha I., Sultan M.T., Randhawa M.A., Saeed F., Ahmed W. (2013). Black Pepper and Health Claims: A Comprehensive Treatise. Crit. Rev. Food Sci. Nutr..

[2] Wattanathorn J., Chonpathompikunlert P., Muchimapura S., Priprem A., Tankamnerdthai O. (2008). Piperine, the Potential Functional Food for Mood and Cognitive Disorders. Food Chem. Toxicol..

[3] Shityakov S., Bigdelian E., Hussein A.A., Hussain M.B., Tripathi Y.C., Khan M.U., Shariati M.A. (2019). Phytochemical and Pharmacological Attributes of Piperine: A Bioactive Ingredient of Black Pepper. Eur. J. Med. Chem..

[4] Mohanraj V., Aravindan B., Jayaprakash C., Thenmozhi M. (2014). In silico drug design and extraction of piperine an inhibitor for fernesyltransferase in *Cryptococcus neoformans*. World J. Pharm. Res..

[5] Chonpathompikunlert P., Yoshitomi T., Han J., Isoda H., Nagasaki Y. (2011). The Use of Nitroxide Radical-Containing Nanoparticles Coupled with Piperine to Protect Neuroblastoma SH-SY5Y Cells from Aβ-Induced Oxidative Stress. Biomaterials.

[6] Chonpathompikunlert P., Wattanathorn J., Muchimapura S. (2010). Piperine, the Main Alkaloid of Thai Black Pepper, Protects against Neurodegeneration and Cognitive Impairment in Animal Model of Cognitive Deficit like Condition of Alzheimer’s Disease. Food Chem. Toxicol..

[7] Liu J., Chen M., Wang X., Wang Y., Duan C., Gao G., Lu L., Wu X., Wang X., Yang H. (2016). Piperine Induces Autophagy by Enhancing Protein Phosphotase 2A Activity in a Rotenone-Induced Parkinson’s Disease Model. Oncotarget.

[8] Stojanović-Radić Z., Pejčić M., Dimitrijević M., Aleksić A., V. Anil Kumar N., Salehi B., C. Cho W., Sharifi-Rad J. (2019). Piperine-A Major Principle of Black Pepper: A Review of Its Bioactivity and Studies. Appl. Sci..

[9] Sriwiriyajan S., Tedasen A., Lailerd N., Boonyaphiphat P., Nitiruangjarat A., Deng Y., Graidist P. (2016). Anticancer and Cancer Prevention Effects of Piperine-Free Piper Nigrum Extract On. Cancer Prev. Res..

[10] Rather R.A., Bhagat M. (2018). Cancer Chemoprevention and Piperine: Molecular Mechanisms and Therapeutic Opportunities. Front. Cell Dev. Biol..

[11] Tiwari A., Mahadik K.R., Gabhe S.Y. (2020). Piperine: A Comprehensive Review of Methods of Isolation, Purification, and Biological Properties. Med. Drug Discov..

[12] Alshehri S., Haq N., Shakeel F. (2018). Solubility, Molecular Interactions and Mixing Thermodynamic Properties of Piperine in Various Pure Solvents at Different Temperatures. J. Mol. Liq..

[13] Hashimoto K., Yaoi T., Koshiba H., Yoshida T., Maoka T., Fujiwara Y., Yamamoto Y., Mori K. (1996). Photochemical Isomerization of Piperine, a Pungent Constituent in Pepper. Food Sci. Technol. Int. Tokyo.

[14] Carneiro S.B., Costa Duarte F.Í., Heimfarth L., Siqueira Quintans J.d.S., Quintans-Júnior L.J., Veiga Júnior V.F.d., Neves de Lima Á.A. (2019). Cyclodextrin^−^Drug Inclusion Complexes: In Vivo and In Vitro Approaches. Int. J. Mol. Sci..

[15] Cheirsilp B., Rakmai J. (2017). Inclusion Complex Formation of Cyclodextrin with Its Guest and Their Applications. Biol. Eng. Med..

[16] Matencio A., Caldera F., Cecone C., López-Nicolás J.M., Trotta F. (2020). Cyclic Oligosaccharides as Active Drugs, an Updated Review. Pharmaceuticals.

[17] Gadade D.D., Pekamwar S.S. (2020). Cyclodextrin Based Nanoparticles for Drug Delivery and Theranostics. Adv. Pharm. Bull..

[18] Ezawa T., Inoue Y., Tunvichien S., Suzuki R., Kanamoto I. (2016). Changes in the Physicochemical Properties of Piperine/Cyclodextrin Due to the Formation of Inclusion Complexes. Int. J. Med. Chem..

[19] Ezawa T., Inoue Y., Murata I., Takao K., Sugita Y., Kanamoto I. (2018). Characterization of the Dissolution Behavior of Piperine/Cyclodextrins Inclusion Complexes. AAPS PharmSciTech.

[20] Ezawa T., Inoue Y., Murata I., Takao K., Sugita Y., Kanamoto I. (2019). Evaluation of the Molecular State of Piperine in Cyclodextrin Complexes by Near-Infrared Spectroscopy and Solid-State Fluorescence Measurements. Int. J. Med. Chem..

[21] Quilaqueo M., Millao S., Luzardo-Ocampo I., Campos-Vega R., Acevedo F., Shene C., Rubilar M. (2019). Inclusion of Piperine in β-Cyclodextrin Complexes Improves Their Bioaccessibility and in Vitro Antioxidant Capacity. Food Hydrocoll..

[22] Debnath S., Mishra J. (2020). Understanding the Intrinsic Fluorescence of Piperine in Microheterogeneous Media: Partitioning and Loading Studies. New J. Chem..

[23] Ezawa T., Inagaki Y., Kashiwaba K., Matsumoto N., Moteki H., Murata I., Inoue Y., Kimura M., Ogihara M., Kanamoto I. (2021). Solubility of Piperine and Its Inclusion Complexes in Biorelevant Media and Their Effect on Attenuating Mouse Ileum Contractions. ACS Omega.

[24] Liu K., Liu H., Li Z., Li W., Li L. (2020). In Vitro Dissolution Study on Inclusion Complex of Piperine with Ethylenediamine-β-Cyclodextrin. J. Incl. Phenom. Macrocycl. Chem..

[25] Hatziagapiou K., Bethanis K., Koniari E., Christoforides E., Nikola O., Andreou A., Mantzou A., Chrousos G.P., Kanaka-Gantenbein C., Lambrou G.I. (2022). Biophysical Studies and In Vitro Effects of Tumor Cell Lines of Cannabidiol and Its Cyclodextrin Inclusion Complexes. Pharmaceutics.

[26] Higuchi T., Connors K.A. (1965). Phase Solubility Techniques. Adv. Anal. Chem. Instrum..

[27] Sheldrick G.M. (2013). SAINT.

[28] Sheldrick G.M. (2012). SADABS.

[29] Sheldrick G.M. (2012). TWINABS.

[30] Sheldrick G.M. (2010). Experimental Phasing with *SHELXC*/*D*/*E*: Combining Chain Tracing with Density Modification. Acta Crystallogr. Sect. D.

[31] Sheldrick G.M. (2015). Crystal Structure Refinement with *SHELXL*. Acta Crystallogr. Sect. C.

[32] Hübschle C.B., Sheldrick G.M., Dittrich B. (2011). *ShelXle*: A Qt Graphical User Interface for *SHELXL*. J. Appl. Crystallogr..

[33] Schüttelkopf A.W., van Aalten D.M.F. (2004). *PRODRG*: A Tool for High-Throughput Crystallography of Protein–Ligand Complexes. Acta Crystallogr. Sect. D Biol. Crystallogr..

[34] Thorn A., Dittrich B., Sheldrick G.M. (2012). Enhanced Rigid-Bond Restraints. Acta Crystallogr. Sect. A: Found. Crystallogr..

[35] Macrae C.F., Bruno I.J., Chisholm J.A., Edgington P.R., McCabe P., Pidcock E., Rodriguez-Monge L., Taylor R., van de Streek J., Wood P.A. (2008). *Mercury* CSD 2.0–New Features for the Visualization and Investigation of Crystal Structures. J. Appl. Crystallogr..

[36] Schrödinger, LLC (2015). The PyMOL Molecular Graphics System, Version 1.8.

[37] Dolomanov O.V., Bourhis L.J., Gildea R.J., Howard J.A.K., Puschmann H. (2009). *OLEX2*: A Complete Structure Solution, Refinement and Analysis Program. J. Appl. Crystallogr..

[38] Trott O., Olson A.J. (2009). AutoDock Vina: Improving the Speed and Accuracy of Docking with a New Scoring Function, Efficient Optimization, and Multithreading. J. Comput. Chem..

[39] Jadhav P. (2021). Piperine-Hydroxy Acid-Cyclodextrin Inclusion Complexes; Antioxidant, Antiinflammatory, and Stability Studies: PART II. AJP.

[40] Pedretti A., Mazzolari A., Gervasoni S., Fumagalli L., Vistoli G. (2021). The VEGA Suite of Programs: An Versatile Platform for Cheminformatics and Drug Design Projects. Bioinformatics.

[41] Gordon M.S., Schmidt M.W., Dykstra C.E., Frenking G., Kim K.S., Scuseria G.E. (2005). Chapter 41-Advances in Electronic Structure Theory: GAMESS a Decade Later. Theory and Applications of Computational Chemistry.

[42] Salomon-Ferrer R., Case D.A., Walker R.C. (2012). An Overview of the Amber Biomolecular Simulation Package. Wiley Interdiscip. Rev. Comput. Mol. Sci..

[43] Cezard C., Trivelli X., Aubry F., Djedaini-Pilard F., Dupradeau F.-Y. (2011). Molecular Dynamics Studies of Native and Substituted Cyclodextrins in Different Media: 1. Charge Derivation and Force Field Performances. Phys. Chem Chem Phys..

[44] Kirschner K.N., Yongye A.B., Tschampel S.M., Gonzalez-Outeirino J., Daniels C.R., Foley B.L., Woods R.J. (2008). GLYCAM06: A Generalizable Biomolecular Force Field. Carbohydrates. J. Comput. Chem..

[45] Mark P., Nilsson L. (2001). Structure and Dynamics of the TIP3P, SPC, and SPC/E Water Models at 298 K. J. Phys. Chem. A.

[46] Roe D.R., Cheatham T.E. (2013). 3rd PTRAJ and CPPTRAJ: Software for Processing and Analysis of Molecular Dynamics Trajectory Data. J. Chem. Theory Comput..

[47] Humphrey W., Dalke A., Schulten K. (1996). VMD: Visual Molecular Dynamics. J. Mol. Graph..

[48] Genheden S., Ryde U. (2015). The MM/PBSA and MM/GBSA Methods to Estimate Ligand-Binding Affinities. Expert Opin. Drug Discov..

[49] Miller B.R., McGee T.D.J., Swails J.M., Homeyer N., Gohlke H., Roitberg A.E. (2012). MMPBSA.Py: An Efficient Program for End-State Free Energy Calculations. J. Chem. Theory Comput..

[50] Wang E., Sun H., Wang J., Wang Z., Liu H., Zhang J.Z.H., Hou T. (2019). End-Point Binding Free Energy Calculation with MM/PBSA and MM/GBSA: Strategies and Applications in Drug Design. Chem. Rev..

[51] Wang J., Hou T., Xu X. (2006). Recent Advances in Free Energy Calculations with a Combination of Molecular Mechanics and Continuum Models. Curr. Comput. -Aided Drug Des..

[52] Gilson M.K., Honig B. (1988). Calculation of the Total Electrostatic Energy of a Macromolecular System: Solvation Energies, Binding Energies, and Conformational Analysis. Proteins Struct. Funct. Bioinform..

[53] Loftsson T., Másson M., Sigurjónsdóttir J.F. (1999). Methods to Enhance the Complexation Efficiency of Cylodextrins. S.T.P. Pharma Sci..

[54] Ceborska M. (2016). Structural Investigation of the β-Cyclodextrin Complexes with Linalool and Isopinocampheol–Influence of Monoterpenes Cyclicity on the Host–Guest Stoichiometry. Chem. Phys. Lett..

[55] Ogawa N., Nagase H., Loftsson T., Endo T., Takahashi C., Kawashima Y., Ueda H., Yamamoto H. (2017). Crystallographic and Theoretical Studies of an Inclusion Complex of β-Cyclodextrin with Fentanyl. Int. J. Pharm..

[56] Bethanis K., Christoforides E., Tsorteki F., Fourtaka K., Mentzafos D. (2018). Structural Studies of the Inclusion Compounds of α-Naphthaleneacetic Acid in Heptakis(2,6-Di-O-Methyl)-β-Cyclodextrin and Heptakis(2,3,6-Tri-O-Methyl)-β-Cyclodextrin by X-Ray Crystallography and Molecular Dynamics. J. Incl. Phenom. Macrocycl. Chem..

[57] Christoforides E., Fourtaka K., Andreou A., Bethanis K. (2020). X-ray Crystallography and Molecular Dynamics Studies of the Inclusion Complexes of Geraniol in β-Cyclodextrin, Heptakis (2,6-Di-O-Methyl)-β-Cyclodextrin and Heptakis (2,3,6-Tri-O-Methyl)-β-Cyclodextrin. J. Mol. Struct..

[58] Bonnet P., Jaime C., Morin-Allory L. (2001). α-, β-, and γ-Cyclodextrin Dimers. Molecular Modeling Studies by Molecular Mechanics and Molecular Dynamics Simulations. J. Org. Chem..

[59] Bethanis K., Christoforides E., Andreou A., Eliopoulos E. (2022). Molecular Symmetry of Permethylated β-Cyclodextrins upon Complexation. Symmetry.

[60] Alshehri S., Imam S.S., Hussain A., Altamimi M.A. (2020). Formulation of Piperine Ternary Inclusion Complex Using β CD and HPMC: Physicochemical Characterization, Molecular Docking, and Antimicrobial Testing. Processes.

[61] Saokham P., Muankaew C., Jansook P., Loftsson T. (2018). Solubility of Cyclodextrins and Drug/Cyclodextrin Complexes. Molecules.

[62] Loftsson T., Hreinsdóttir D., Másson M. (2005). Evaluation of Cyclodextrin Solubilization of Drugs. Int. J. Pharm..

[63] Loftsson T., Hreinsdóttir D., Másson M. (2007). The Complexation Efficiency. J. Incl. Phenom. Macrocycl. Chem..

[64] Wenz G., Han B.-H., Müller A. (2006). Cyclodextrin Rotaxanes and Polyrotaxanes. Chem. Rev..

[65] Anconi C., Soares Nascimento Junior C., Almeida W., Santos H. (2008). Theoretical Study of α-CD Based [3] Pseudorotaxanes: The Role Played by Threadlike Polymer on the Stability of Cyclodextrin Dimers. J. Braz. Chem. Soc. -JBCS.

[66] Christoforides E., Papaioannou A., Bethanis K. (2018). Crystal Structure of the Inclusion Complex of Cholesterol in β-Cyclodextrin and Molecular Dynamics Studies. Beilstein J. Org. Chem..

[67] Dos Santos C., Buera M.P., Mazzobre M.F. (2011). Phase Solubility Studies and Stability of Cholesterol/Beta-Cyclodextrin Inclusion Complexes. J. Sci Food Agric..

[68] Imam S.S., Alshehri S., Alzahrani T.A., Hussain A., Altamimi M.A. (2020). Formulation and Evaluation of Supramolecular Food-Grade Piperine HP β CD and TPGS Complex: Dissolution, Physicochemical Characterization, Molecular Docking, In Vitro Antioxidant Activity, and Antimicrobial Assessment. Molecules.

