# Supplementary material for: Structural Studies of Piperine Inclusion Complexes in Native and Derivative β-Cyclodextrins
Source: Biomolecules. 2022 Nov 26;12(12):1762. doi: 10.3390/biom12121762 (PMC9775453; doi:10.3390/biom12121762)
Supplement: Supplementary file 1 [file biomolecules-12-01762-s001.zip › biomolecules-1966573-supplementary.pdf]

biomolecules

MDPI

*Supplementary material*

# Structural Studies of Piperine Inclusion Complexes in Native and Derivative β-Cyclodextrins

**Elias Christoforides [1], Athena Andreou [2], Andreas Papaioannou [1] and Kostas Bethanis [1,*]**

[1] Physics Laboratory, Department of Biotechnology, Agricultural University of Athens, 75 Iera Odos, Athens 11855, Greece
[2] Genetics Laboratory, Department of Biotechnology, Agricultural University of Athens, 75 Iera Odos, Athens 11855, Greece
* Correspondence: kbeth@aua.gr; Tel.:+302105294211

## Contents

**Figure S1**. The solubility profile of pure piperine in an equimolar water: methanol solution (n=3) .................................. 2

**Figure S2.** The distances and angles as defined in Tables S2-S4. In particular the following are presented: (a) d=O4n…O4(n+1) distances; (b) dK=distances of the approximate center K of the O4n heptagon from the O4n atoms; (c) dev=deviations of the O4n atoms from their least-squares plane; (d) φ =O4(n-1)…O4n … O4(n+1) angles; (e) φK=O4n… K…O4(n+1) angles. All distances are given in Å and angles in (°). .......................................................................... 4

**Figure S3.** RMSD to first frame vs time plots for the 4 host and two guest molecules in the supramolecular ensemble of PN/β-CD .......................................................................................................................................... 5

**Figure S4.** Number of H-bonds between hydroxyls of the wide rims of adjacent PN/*β*-CD complex units within the same channel. ....................................................................................................................................... 5

**Figure S5.** H-H close contacts between guest's (PN1) H14, H17 and H18 atoms and host's (β-CD3) H3 atoms of specific glucose residues (G1, G2, G3, G4, G5 and G6). ........................................................................................ 6

**Figure S6**. H-H close contacts between guest's (PN1) H14, H17 and H18 atoms and host's (β-CD3) H5 atoms of residues G1, G2, G3, G4, G5 and G6. ........................................................................................................ 7

**Figure S7.** Plots of contacts between PN1 and PN2 guests and β-CD3/β-CD4 and β-CD1/β-CD2 host duets, respectively in PN/*β*-CD complex case. .................................................................................................................. 8

**Figure S8.** Number of H-bonds between hosts and guests in PN/β-CD complex case........................................................ 8

**Table S1:** Main intramolecular interactions present in the crystal structures of PN/β-CD, PN/DM-β-CD and PN/TM-β-CD inclusion complexes ..................................................................................................................... 9

**Table S2.** Conformational characteristics of the two β-CD host molecules in the PN/β-CD structure ............................. 12

**Table S3.** Conformational characteristics of the DM-β-CD host molecule in the PN/DM-β-CD structure....................... 13

**Table S4.** Conformational characteristics of the two TM-β-CD host molecules in the PN/TM-β-CD structure .............. 13

**Table S5.** H bond analysis for the two encapsulated PN molecules (PN_1 and PN_2) in their complex with four *β*-CDs during the 12 ns MD simulation. ............................................................................................................. 15

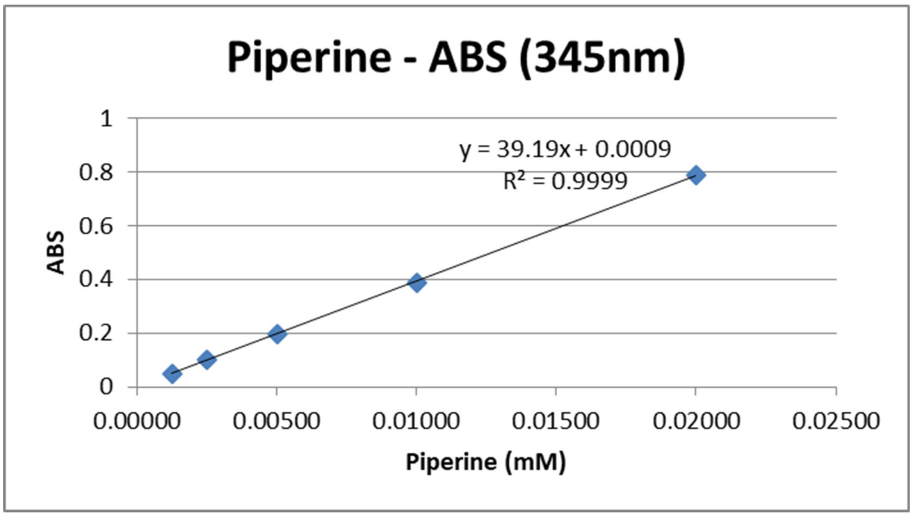


**Figure S1.** The solubility profile of pure piperine in an equimolar water: methanol solution (n=3).

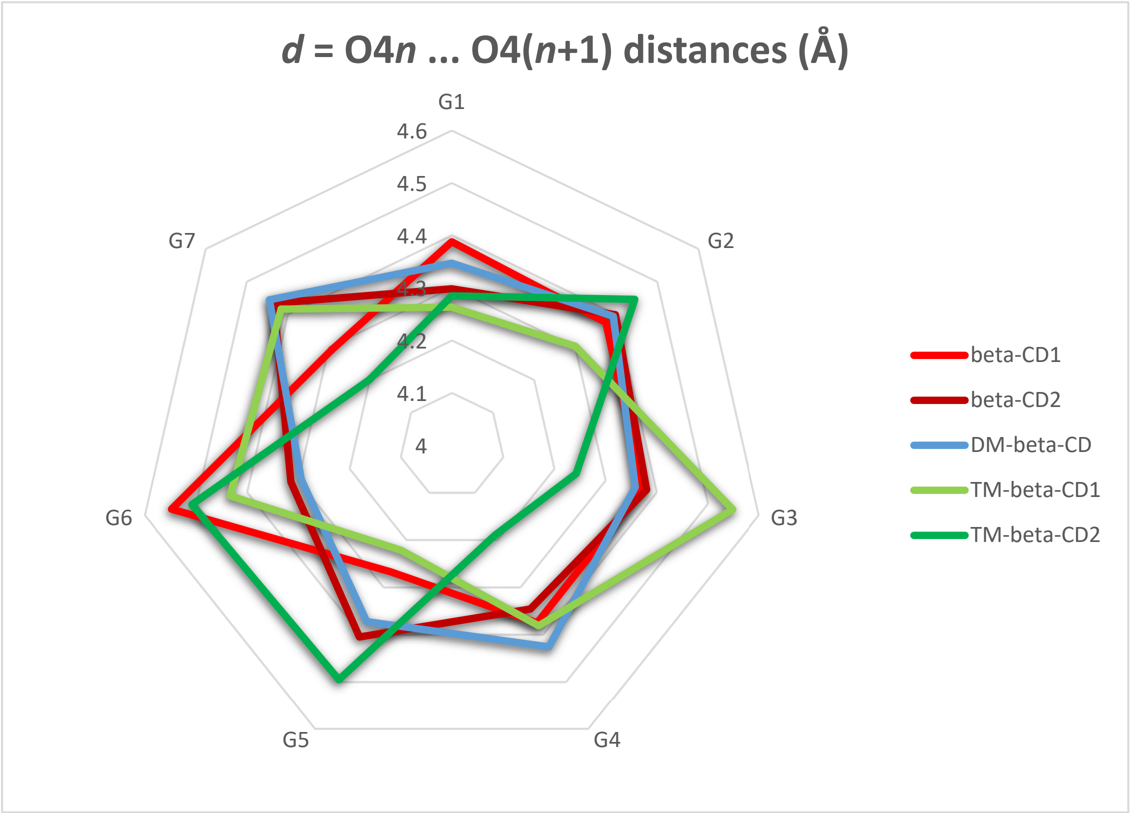


(a)

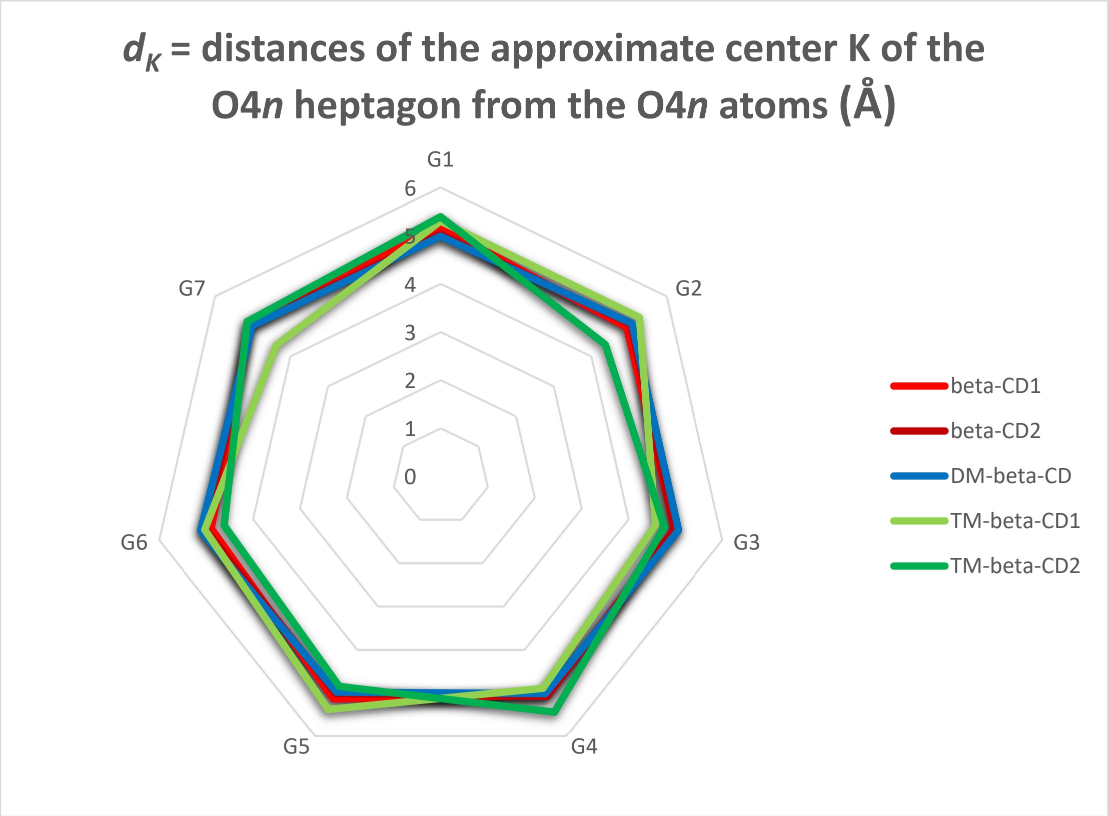

$d_K$ = distances of the approximate center K of the O4*n* heptagon from the O4*n* atoms (Å)
G1
G2
G3
G4
G5
G6
G7
6
5
4
3
2
1
0
beta-CD1
beta-CD2
DM-beta-CD
TM-beta-CD1
TM-beta-CD2


(b)

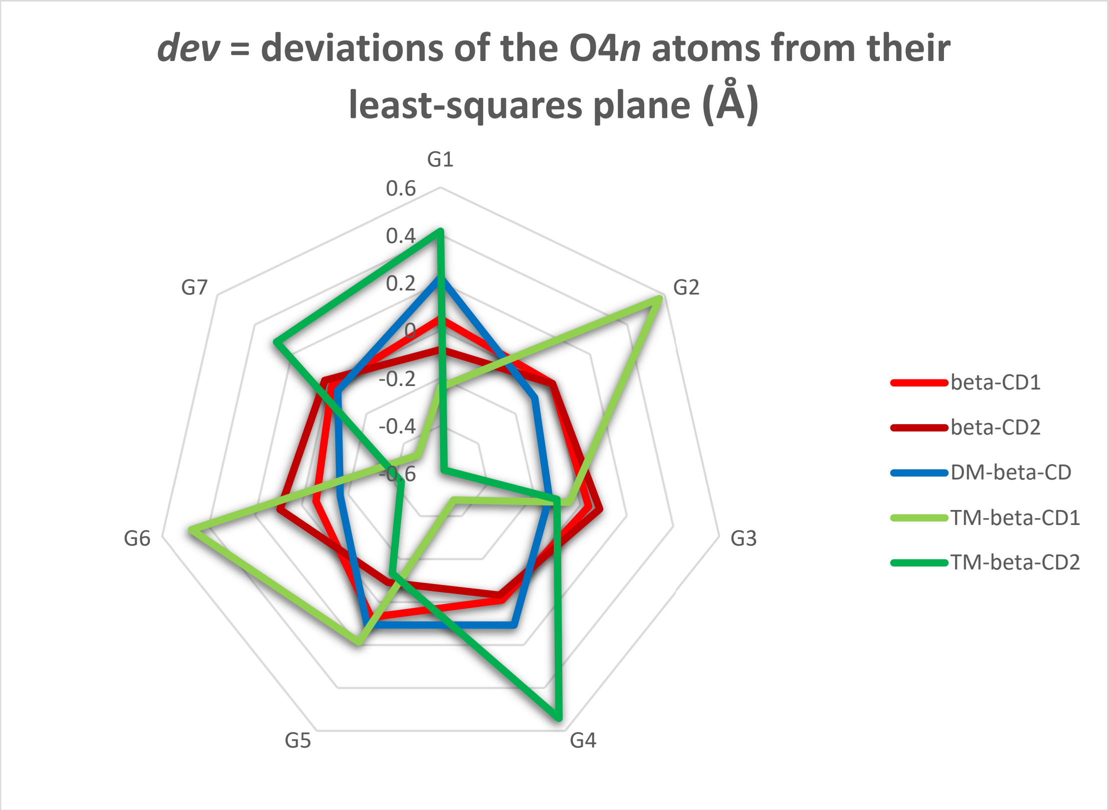

*dev* = deviations of the O4*n* atoms from their least-squares plane (Å)
G1
G2
G3
G4
G5
G6
G7
0.6
0.4
0.2
0
-0.2
-0.4
-0.6
beta-CD1
beta-CD2
DM-beta-CD
TM-beta-CD1
TM-beta-CD2


(c)

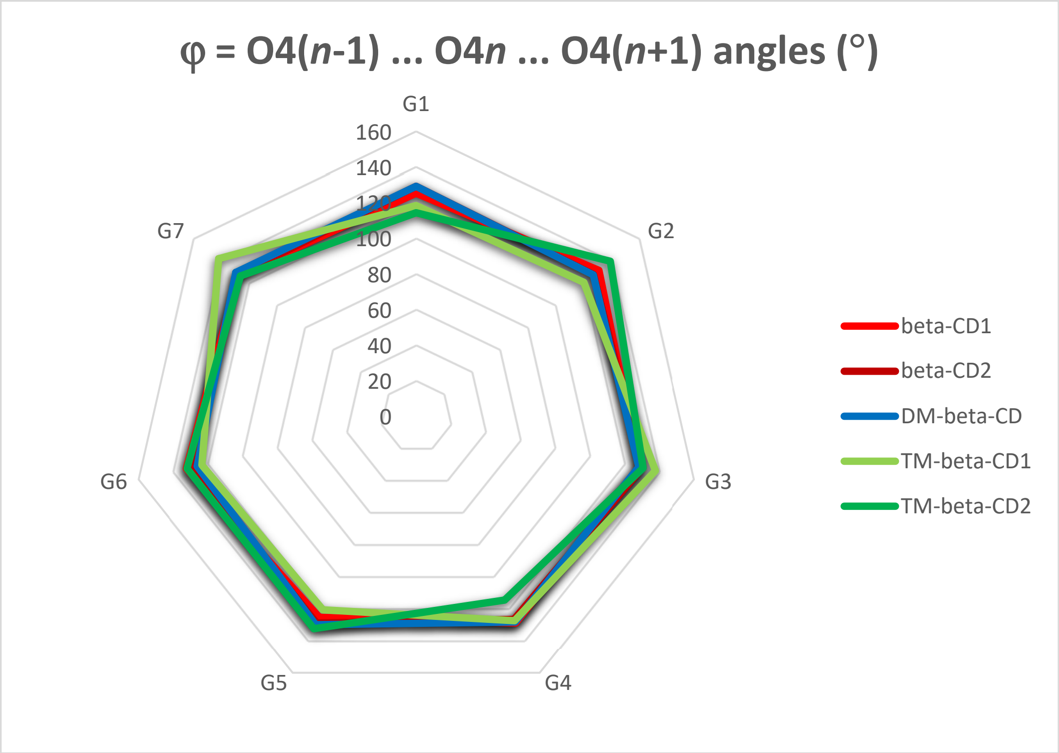


(d)

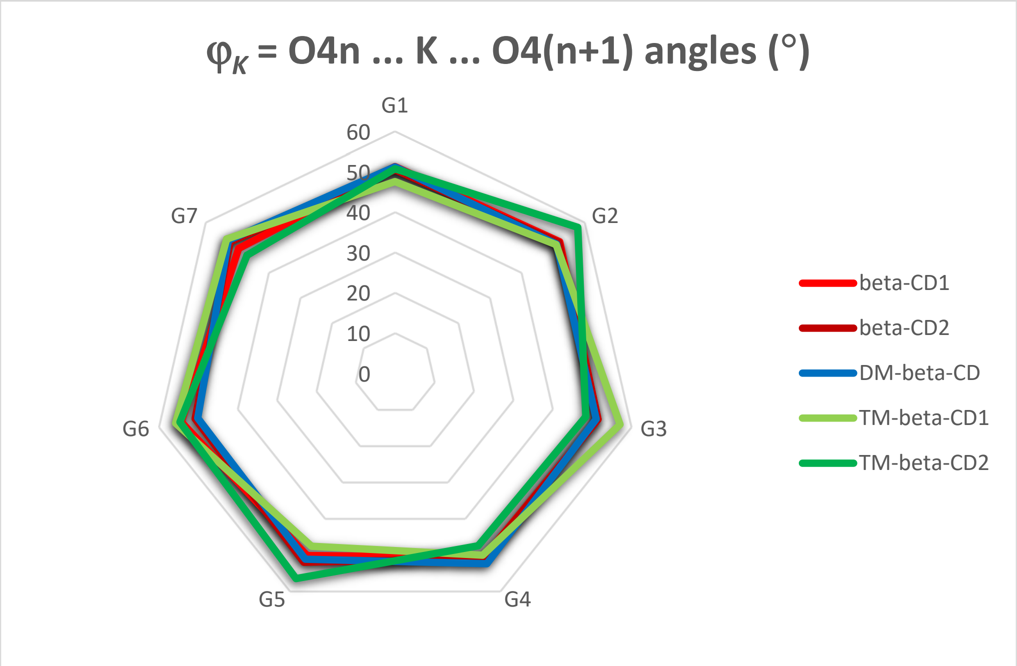


(e)

**Figure S2.** The distances and angles as defined in Tables S2-S4. In particular the following are presented: (a) d=O4n…O4(n+1) distances; (b) dK=distances of the approximate center K of the O4n heptagon from the O4n atoms; (c) dev=deviations of the O4n atoms from their least-squares plane; (d) φ =O4(n-1)…O4n … O4(n+1) angles; (e) φK=O4n…K…O4(n+1) angles. All distances are given in Å and angles in (°).

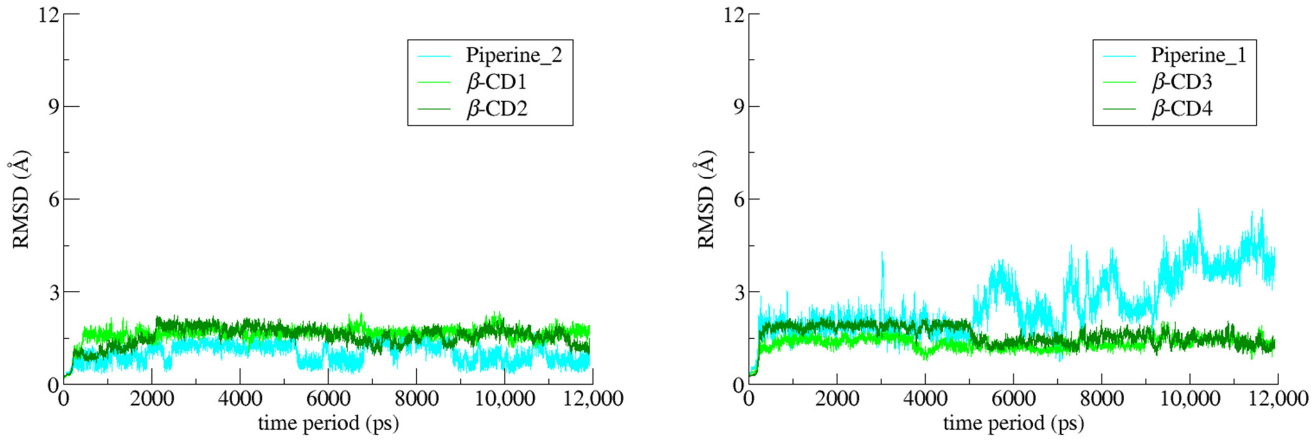


**Figure S3.** RMSD to first frame vs time plots for the 4 host and two guest molecules in the supramolecular ensemble of PN/*β*-CD.

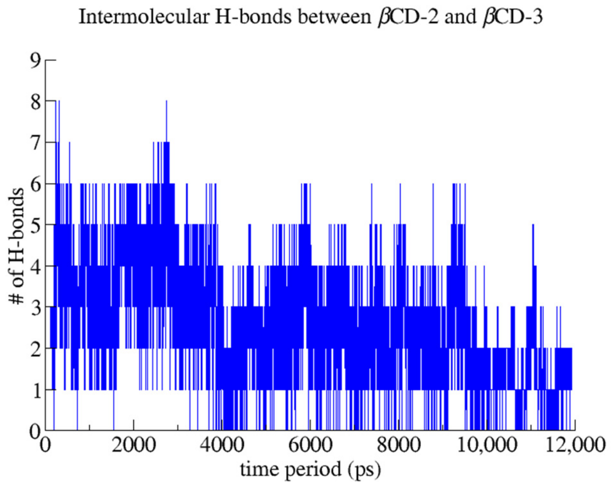


**Figure S4.** Number of H-bonds between hydroxyls of the wide rims of adjacent PN/*β*-CD complex units within the same channel.

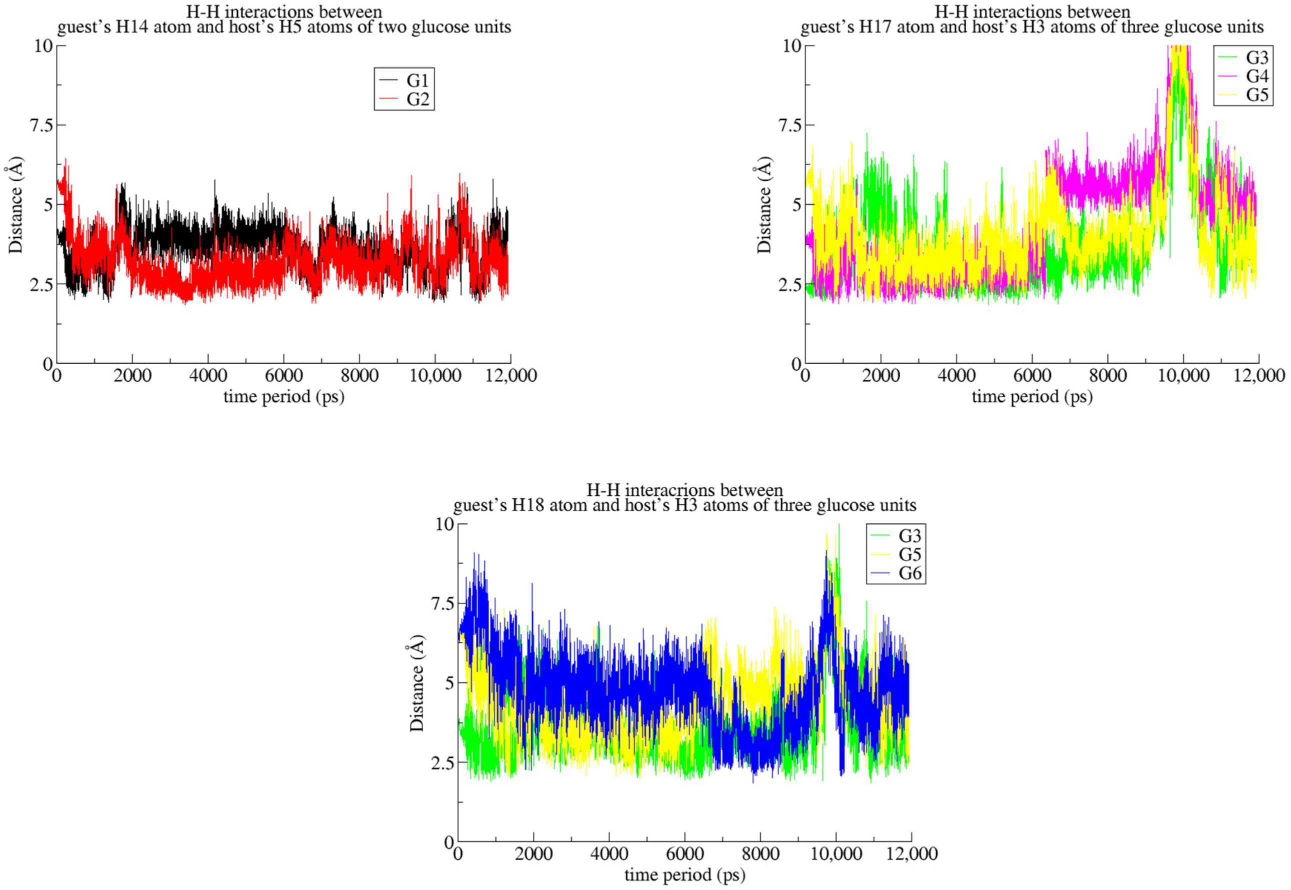


**Figure S5.** H-H close contacts between guest's (PN1) H14, H17 and H18 atoms and host's (*β*-CD3) H3 atoms of specific glucose residues (G1, G2, G3, G4, G5 and G6).

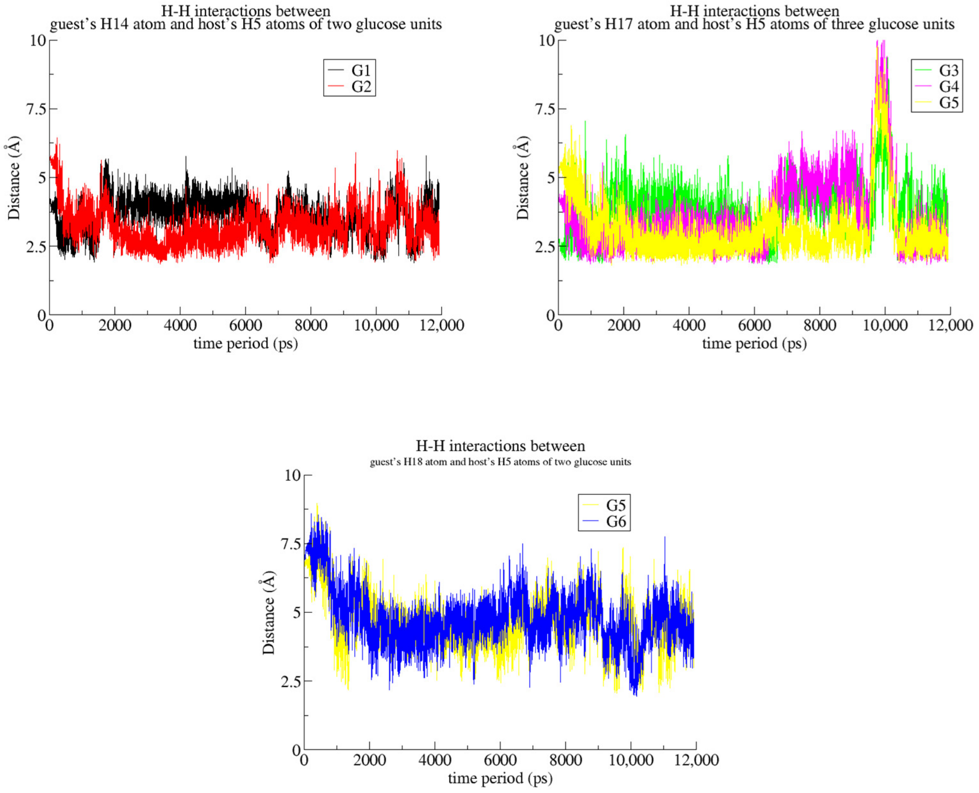


**Figure S6**. H-H close contacts between guest's (PN1) H14, H17 and H18 atoms and host's (β-CD3) H5 atoms of residues G1, G2, G3, G4, G5 and G6.

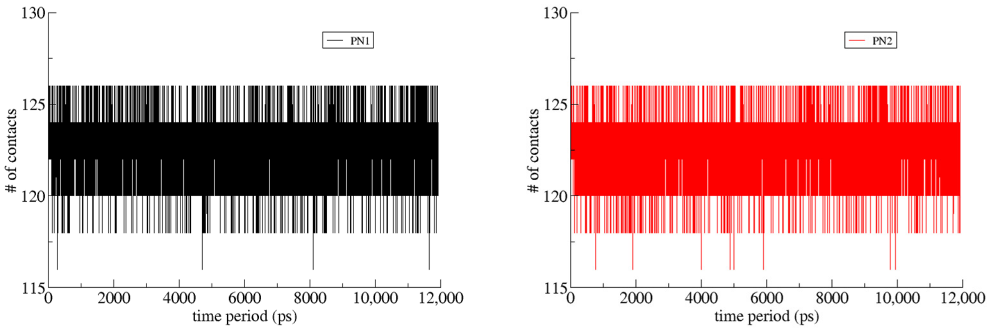


**Figure S7.** Plots of contacts between PN1 and PN2 guests and *β*-CD3/*β*-CD4 and *β*-CD1/*β*-CD2 host duets, respectively in PN/*β*-CD complex case.

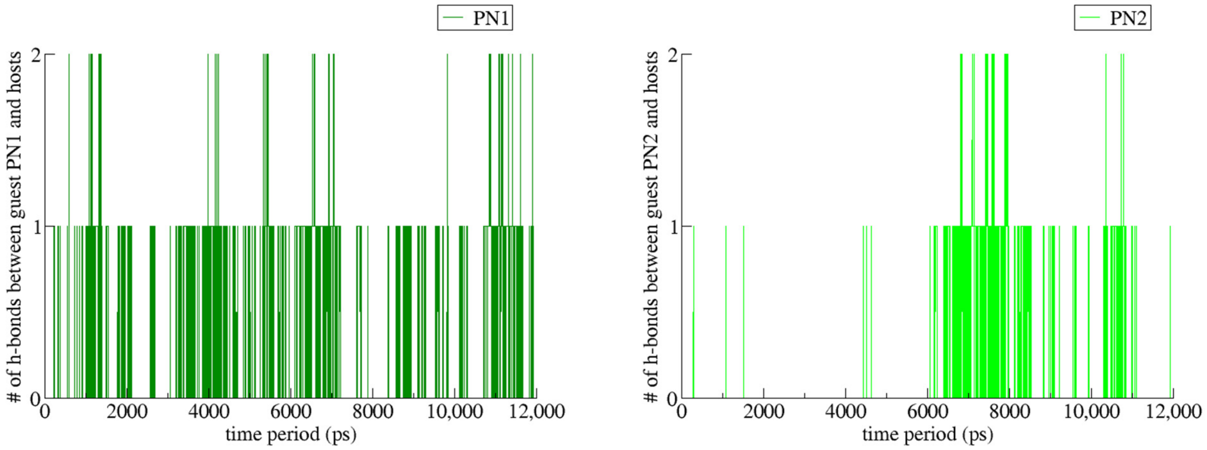


**Figure S8.** Number of H-bonds between hosts and guests in PN/*β*-CD complex case.

**Table S1:** Main intramolecular interactions present in the crystal structures of PN/*β*-CD, PN/DM-*β*-CD and PN/TM-*β*-CD inclusion complexes.

## a1. Main Hydrogen Bonds in PN/*β*-CD case

| Donor---Hydrogen...Acceptor | Don--Hyd [Å] | Hyd--Acc [Å] | Don--Acc [Å] | D--H-----A |
|---|---|---|---|---|
| **O31A---H31L...O22A** | 0.84 | 2.08 | 2.868 | 157.2° |
| **O61A---H61O...OW5** | 0.84 | 2.05 | 2.853 | 159.6° |
| **O22A---H22D...OW3P_a** | 0.84 | 1.94 | 2.738 | 159.4° |
| **O23A---H23B...OW18_a** | 0.84 | 1.75 | 2.580 | 170.0° |
| **O33A---H33N...OW6** | 0.84 | 1.95 | 2.787 | 178.4° |
| **O63A---H63I...OW1** | 0.84 | 1.89 | 2.727 | 175.0° |
| **O34A---H34J...O25A** | 0.84 | 1.91 | 2.743 | 172.5° |
| **O64A---H64U...OW13** | 0.84 | 2.05 | 2.860 | 163.4° |
| **O36A---H36B...O27A** | 0.84 | 1.85 | 2.683 | 173.2° |
| **O66A---H66X...OW9** | 0.84 | 2.48 | 3.277 | 158.3° |
| **O27A---H27C...OW8** | 0.84 | 1.80 | 2.637 | 172.8° |
| **O37A---H37B...O21A** | 0.84 | 2.03 | 2.854 | 167.7° |
| **O67A---H67K...O61B** | 0.84 | 1.88 | 2.721 | 175.0° |
| **O31B---H31C...O22B** | 0.84 | 2.03 | 2.780 | 147.8° |
| **O61B---H61E...OW16** | 0.84 | 1.93 | 2.691 | 149.6° |
| **O62B---H62E...OW9** | 0.84 | 2.19 | 2.874 | 139.2° |
| **O23B---H23C...O32B** | 0.84 | 1.96 | 2.772 | 162.7° |
| **O34B---H34C...O25B** | 0.84 | 1.98 | 2.814 | 171.1° |
| **O64B---H64E...OW13** | 0.84 | 2.04 | 2.842 | 159.1° |
| **O35B---H35D...O26B** | 0.84 | 2.02 | 2.843 | 167.0° |
| **O66B---H66E...O63A** | 0.84 | 1.94 | 2.768 | 170.9° |
| **O27B---H27D...O36B** | 0.84 | 1.92 | 2.739 | 164.4° |
| **O37B---H37C...O21B** | 0.84 | 2.04 | 2.770 | 145.5° |

## a2. Other host – guest or guest-guest interactions in PN/*β*-CD case

| Atoms | Distance [Å] | Angle (°) | Type of interaction |
|---|---|---|---|
| **PN1** | | | |
| **HAEB – OAC(x,y,-1+z)**<br>**CAE-HAEB – OAC(x,y,-1+z)** | 1.889 | 162.21° | C-H..O interaction |
| **HAEB – HAUB(x,y,-1+z)** | 2.285 | | H-H interaction |
| **HAFA – H53B** | 2.344 | | H-H interaction |
| **HAFB –H52B** | 2.349 | | H-H interaction |
| **HAIA-H56B** | 2.239 | | H-H interaction |
| **HAIA-O46B**<br>**CAI- HAIA-O46B** | 2.682 | 153.11° | C-H…O interaction |
| **HAKB-O65A**<br>**CAK- HAKB-O65A** | 2.583 | 141.31° | C-H…O interaction |
| **HAR-H53A** | 2.338 | | H-H interaction |
| **HAS-H32A** | 2.327 | | H-H interaction |
| **H36A-OAB**<br>**C36A- H36A-OAB** | 2.457 | 125.92° | C-H…O interaction |
| **H37A-OAB**<br>**C37A- H37A-OAB** | 2.637 | 151.87° | C-H…O interaction |
| **HAUA-H32C(x,y,1+z)** | 2.318 | | H-H interaction |
| **HAUA-O32B(x,y,1+z)**<br>**CAU-HAUA-O32B(x,y,1+z)** | 2.389 | 159.76° | C-H…O interaction |
| **HAUB-H33B(x,y,1+z)** | 2.130 | | H-H interaction |
| **PN2** | | | |
| **HAUA-HAEA(x,y,-1+z)** | 2.256 | | H-H interaction |
| **HAUA-HAIB(x,y,-1+z)** | 1.958 | | H-H interaction |

| | | | |
|---|---|---|---|
| **HAT-H34B** | 2.091 | | H-H interaction |
| **HAR-H57B** | 2.184 | | H-H interaction |
| **HAKB-H54A** | 2.311 | | H-H interaction |
| **HAIA-H55A** | 2.383 | | H-H interaction |

## b. Other host – guest interactions in PN/DM-*β*-CD case

| Atoms | Distance [Å] | Angle (°) | Type of interaction |
|---|---|---|---|
| **HAT-O41**<br>**C-HAT-O41** | 2.646 | 175.53° | C-H…O interaction |
| **HAP-O44**<br>**C-HAP-O44** | 2.537 | 157.76° | C-H…O interaction |

## c. Other host – guest interactions in PN/TM-*β*-CD case

| Atoms | Distance [Å] | Angle (°) | Type of interaction |
|---|---|---|---|
| **HAS – H55B** | 2.243 | | H-H interaction |
| **HAQ – H62C** | 2.294 | | H-H interaction |
| **HAI2 – H71F** | 1.954 | | H-H interaction |
| **HAF1 –H54A** | 2.225 | | H-H interaction |
| **HAF2-H97B** | 2.29 | | H-H interaction |
| **HAE1-H57A** | 2.182 | | H-H interaction |
| **HAE1-O76A**<br>**C- HAE1-O76A** | 2.656 | 150.03° | C-H…O interaction |

**Table S2.** Conformational characteristics of the two *β*-CD host molecules in the PN/*β*-CD structure.

| Glucose unit | ($n$ = 1) | ($n$ = 2) | ($n$ = 3) | ($n$ = 4) | ($n$ = 5) | ($n$ = 6) | ($n$ = 7) |
|---|---|---|---|---|---|---|---|
| **Host A** | | | | | | | |
| $d$ | 4.3881(5) | 4.3757(5) | 4.3730(5) | 4.3757(4) | 4.2678(5) | 4.5479(5) | 4.2921(4) |
| $\varphi$ | 125.293(5) | 131.385(6) | 128.580(6) | 128.873(6) | 125.446(8) | 132.485(5) | 127.797(8) |
| $d_K$ | 5.1833(6) | 4.9509(5) | 4.9852(5) | 5.0623(6) | 5.1439(6) | 4.8944(4) | 5.0548(6) |
| $\varphi_K$ | 51.253(7) | 52.256(5) | 51.593(7) | 50.766(5) | 50.245(7) | 54.373(7) | 49.552(5) |
| *dev* | 0.047316 (6) | 0.00044197 (8) | 0.037446 (4) | -0.0057490 (7) | 0.073491 (9) | -0.062718 (7) | -0.0153357 (18) |
| $\tau$ | 14.452(2) | 7.4592(12) | 9.6548(14) | 10.5058(17) | 8.2666(13) | 1.8711(3) | 9.4593(14) |
| $\tau_1$ | -60.469(8) | -64.306(7) | -71.092(8) | -64.506(8) | 64.944(7) | -62.546(8) | 64.928(7) |
| $C$ | *gg* | *gg* | *gg* | *gg* | *gt* | *gg* | *gt* |
| **Host B** | | | | | | | |
| $d$ | 4.2985(5) | 4.3990(5) | 4.3811(4) | 4.3463(5) | 4.4054(5) | 4.3144(5) | 4.4341(4) |
| $\varphi$ | 129.180(7) | 127.541(5) | 130.321(7) | 126.789(5) | 128.935(7) | 129.166(6) | 127.796(6) |
| $d_K$ | 5.0268(6) | 5.0547(4) | 4.9790(6) | 5.0865(6) | 5.0277(6) | 5.0086(5) | 5.0464(6) |
| $\varphi_K$ | 50.474(7) | 52.000(6) | 51.590(6) | 50.895(7) | 52.073(5) | 50.817(7) | 52.231(5) |
| *dev* | -0.0819913 (10) | 0.00050703 (15) | 0.085127 (10) | -0.029364 (3) | -0.088673 (10) | 0.092489 (11) | 0.021827 (3) |
| $\tau$ | 10.0008(15) | 3.7381(6) | 7.5981 (12) | 11.4532(19) | 7.4648(12) | 11.0313(16) | 8.2194(13) |
| $\tau_1$ | -68.381(6) | -68.695(8) | -61.310(4) | 67.860(6) | -65.368(6) | -64.303(8) | -58.942(7) |
| $C$ | *gg* | *gg* | *gg* | *gt* | *gg* | *gg* | *gg* |

**Table S3.** Conformational characteristics of the DM-β-CD host molecule in the PN/DM-β-CD structure.

| Glucose unit | ($n$ = 1) | ($n$ = 2) | ($n$ = 3) | ($n$ = 4) | ($n$ = 5) | ($n$ = 6) | ($n$ = 7) |
|---|---|---|---|---|---|---|---|
| $d$ | 4.3473(8) | 4.3942(10) | 4.3587(7) | 4.4266(7) | 4.3731(9) | 4.2941(8) | 4.4445(6) |
| $\varphi$ | 129.144(11) | 127.373(12) | 128.276(11) | 128.316(11) | 129.989(10) | 126.278(12) | 129.536(10) |
| $d_K$ | 4.9832(10) | 5.0834(7) | 5.0718(8) | 5.0231(11) | 4.9889(9) | 5.1185(7) | 5.0075(10) |
| $\varphi_K$ | 51.159(11) | 51.279(12) | 51.157(11) | 52.478(12) | 51.254(11) | 50.169(11) | 52.828(12) |
| $dev$ | 0.22027 (3) | -0.094344 (12) | -0.132031 (17) | 0.110000 (14) | 0.109923 (14) | -0.16698 (2) | -0.046837 (6) |
| $\tau$ | +9.8121(15) | +9.0032(14) | +23.329(4) | +14. 060(3) | +10.945(3) | +8.2702(5) | +23.2098(7) |
| $\tau_1$ | -70.635(10) | -67.784(12) | 68.062(11) | -63.526(13) | -64.810(15) | -74.383(15) | -74.956(12) 62.522(9) |
| C | *gg* | *gg* | *gt* | *gg* | *gg* | *gg* | *gg* *gt* |

**Table S4.** Conformational characteristics of the two TM-β-CD host molecules in the PN/TM-β-CD structure.

| Glucose unit | ($n$ = 1) | ($n$ = 2) | ($n$ = 3) | ($n$ = 4) | ($n$ = 5) | ($n$ = 6) | ($n$ = 7) |
|---|---|---|---|---|---|---|---|
| **Host A** | | | | | | | |
| $d$ | 4.2633(3) | 4.3022(2) | 4.5493(3) | 4.3817(2) | 4.2223(3) | 4.4336(3) | 4.4160(3) |
| $\varphi$ | 118.3478(14) | 120.555(4) | 137. 856(3) | 127.560(2) | 120.821(4) | 123.5621(9) | 141.947(3) |
| $d_K$ | 5.2952(3) | 5.2834(3) | 4.5786(3) | 4.9028(3) | 5.3870(4) | 5.0298(2) | 4.3622(3) |
| $\varphi_K$ | 47.533(4) | 51.1182(5) | 57.224(5) | 50.136(2) | 47.672(3) | 55.792(3) | 53.3640 (19) |
| $dev$ | -0.240455 (16) | 0.57472 (4) | -0.045129 (4) | -0.47315 (3) | 0.189997 (13) | 0.47408 (3) | -0.48006 (3) |
| $\tau$ | -14.6415 (13) | +41.661 (4) | +35.710 (3) | -9.3716 (8) | +12.7837 (11) | +24.0637 (18) | +43.631 (3) |
| $\tau_1$ | -81.523(4) 64.8896(18) | -67.492(2) | 73.5725 (10) | -66.665(4) | 73.0633(16) | -72.913(5) | 55.883(3) -79.410(2) |
| C | *gg* *gt* | *gg* | *gt* | *gg* | *gt* | *gg* | *gt* *gg* |

| **Host B** | | | | | | | |
|---|---|---|---|---|---|---|---|
| *d* | 4.2850(3) | 4.4461(3) | 4.2435(3) | 4.1902(2) | 4.4959(3) | 4.5081(2) | 4.2004(3) |
| $\varphi$ | 114.3155(7) | 139.544(3) | 130.8004(9) | 114.720(4) | 132.534(3) | 132.091(2) | 126.245(4) |
| $d_K$ | 5.3900(3) | 4.3776(3) | 4.7664(3) | 5.4501(3) | 4.8580(3) | 4.6181(2) | 5.1408(3) |
| $\varphi_K$ | 50.681(3) | 57.999(2) | 48.517(4) | 47.5417(5) | 56.579(5) | 54.709(2) | 46.893(3) |
| *dev* | 0.41535 (3) | -0.58164 (4) | -0.098670 (8) | 0.54499 (4) | -0.130854 (9) | -0.43059 (3) | 0.281422 (19) |
| $\tau$ | +31.410 (2) | +36.246 (2) | -24.504 (2) | +39.326 (3) | +28.244 (2) | -6.9941 (6) | +14.5944 (13) |
| $\tau_1$ | -75.403(5) | -76.599(3) 86.153(4) | -78.911(3) | 78.903(4) -41.9868 (12) | -59.392(3) | -63.146(5) | -71.4223 (16) |
| *C* | *gg* | *gg* *gt* | *gg* | *gt* *gg* | *gg* | *gg* | *gg* |

*d* = O4*n*…O4(*n*+1) distances;
$\varphi$ = O4(*n*-1)…O4*n* … O4(*n*+1) angles;
$d_K$ = distances of the approximate center K of the O4*n* heptagon from the O4*n* atoms;
$\varphi_K$ = O4*n*… K…O4(*n*+1) angle;
*dev* = deviations of the O-4*n* atoms from their least-squares plane;
$\tau$ = tilt angles between the optimum O4*n* plane and the mean plane atoms O4(*n*-1), C1*n*, C4*n*, O4*n*;
$\tau_1$ = O5*n*-C5*n*-C6*n*-O6*n* torsion angles;
*C* = conformation of the primary methoxy groups
All distances are given in Å and angles in (°).

**Table S5.** H bond analysis for the two encapsulated PN molecules (PN_1 and PN_2) in their complex with four *β*-CDs during the 12 ns MD simulation.

| Acceptor | Donor H | Donor | Frames | Fraction | Avg. Distance | Avg. Angle |
|---|---|---|---|---|---|---|
| PN_1@O1 | 4GA_27@H6O | 4GA_27@O6 | 1162 | 0.0974 | 2.7356 | 161.9263 |
| PN_1@O1 | 4GA_24@H6O | 4GA_24@O6 | 949 | 0.0795 | 2.7436 | 162.7801 |
| PN_1@O1 | 4GA_26@H6O | 4GA_26@O6 | 897 | 0.0752 | 2.7559 | 163.7480 |
| PN_1@O1 | 4GA_25@H6O | 4GA_25@O6 | 520 | 0.0436 | 2.7341 | 161.0260 |
| PN_1@O1 | 4GA_18@H6O | 4GA_18@O6 | 249 | 0.0209 | 2.7544 | 161.6698 |
| PN_1@O1 | 4GA_23@H6O | 4GA_23@O6 | 234 | 0.0196 | 2.7650 | 161.2424 |
| PN_1@O1 | 4GA_19@H6O | 4GA_19@O6 | 51 | 0.0043 | 2.8471 | 155.0095 |
| PN_1@O3 | 4GA_16@H2O | 4GA_16@O2 | 20 | 0.0017 | 2.9227 | 151.3817 |
| PN_1@O3 | 4GA_20@H3O | 4GA_20@O3 | 16 | 0.0013 | 2.8907 | 147.9467 |
| PN_1@O3 | 4GA_18@H2O | 4GA_18@O2 | 11 | 0.0009 | 2.8865 | 151.1731 |
| PN_1@O2 | 4GA_15@H6O | 4GA_15@O6 | 6 | 0.0005 | 2.8191 | 152.5074 |
| PN_1@O2 | 4GA_15@H3O | 4GA_15@O3 | 4 | 0.0003 | 2.8763 | 144.5483 |
| PN_1@O2 | 4GA_20@H3O | 4GA_20@O3 | 2 | 0.0002 | 2.9269 | 160.3391 |
| PN_1@O3 | 4GA_17@H2O | 4GA_17@O2 | 1 | 0.0001 | 2.9866 | 138.4496 |
| PN_2@O1 | 4GA_10@H6O | 4GA_10@O6 | 987 | 0.0827 | 2.7449 | 163.3364 |
| PN_2@O1 | 4GA_4@H6O | 4GA_4@O6 | 563 | 0.0472 | 2.7603 | 157.9697 |
| PN_2@O1 | 4GA_9@H6O | 4GA_9@O6 | 356 | 0.0298 | 2.7420 | 162.8458 |
| PN_2@O1 | 4GA_12@H6O | 4GA_12@O6 | 53 | 0.0044 | 2.7568 | 162.1470 |
| PN_2@O3 | 4GA_4@H6O | 4GA_4@O2 | 21 | 0.0018 | 2.8796 | 150.9538 |
| PN_2@O3 | 4GA_3@H6O | 4GA_3@O2 | 15 | 0.0013 | 2.8895 | 149.6184 |
| PN_2@O1 | 4GA_8@H6O | 4GA_8@O6 | 11 | 0.0009 | 2.8139 | 162.9386 |
| PN_2@O3 | 4GA_6@H6O | 4GA_6@O3 | 9 | 0.0008 | 2.8485 | 148.4055 |
| PN_2@O2 | 4GA_1@H6O | 4GA_1@O6 | 9 | 0.0008 | 2.8752 | 156.8046 |
| PN_2@O3 | 4GA_2@H6O | 4GA_2@O2 | 6 | 0.0005 | 2.8865 | 151.2473 |
| PN_2@O1 | 4GA_11@H6O | 4GA_11@O6 | 2 | 0.0002 | 2.6996 | 150.0678 |
| PN_2@O2 | 4GA_7@H6O | 4GA_7@O2 | 1 | 0.0001 | 2.8591 | 141.4388 |
| PN_2@O3 | 4GA_3@H6O | 4GA_3@O3 | 1 | 0.0001 | 2.9679 | 148.9800 |
